# Supplementary material for: Retroviral Replicating Vector Delivery of miR-PDL1 Inhibits Immune Checkpoint PDL1 and Enhances Immune Responses In Vitro
Source: Mol Ther Nucleic Acids. 2016 Dec 10;6:221–32. doi: 10.1016/j.omtn.2016.11.007 (PMC5363416; doi:10.1016/j.omtn.2016.11.007)
Supplement: Document S1. Figures S1–S6 and Tables S1–S4 [file mmc1.pdf]

OMTN, Volume *6*

Supplemental Information

Retroviral Replicating Vector Delivery of miR-PDL1 Inhibits Immune Checkpoint PDL1 and Enhances Immune Responses In Vitro

Amy H. Lin, Christopher G. Twitty, Ryan Burnett, Andrew Hofacre, Leah A. Mitchell, Fernando Lopez Espinoza, Harry E. Gruber, and Douglas J. Jolly

Supplemental Table 1: Viral titers of RRV of various shRNA configurations produced from transiently transfected 293T cells.

RRV	Titer (TU/mL)	Stdev
**IRES-yCD2 (Toca 511)**	5.48E+06	1.25E+05
**IRES-GFP**	3.30E+06	1.43E+05
**RSV-yCD2**	1.92E+06	1.85E+05
**U6-shGFP1***	2.95E+06	9.69E+04
**U6-shGFP2***	2.11E+06	1.43E+05
**U6-miRGFP1***	2.64E+06	1.08E+05
**U6-miRGFP2***	2.63E+06	8.59E+04
**U6-miRPDL1***	3.99E+06	2.33E+05
**H1-miRPDL1**	5.10E+06	5.48E+05
**RSV-miRPDL1**	1.34E+06	5.85E+04
**RSV-yCD2-miRPDL1**	2.00E+06	1.81E+05
**RSV-yCD2-U6-miRPDL1**	1.71E+06	3.18E+05

*Described as shGFP1, shRGFP2, miRGFP1, miRGFP2, miRPDL1, miRPDL1 in the manuscript.

Supplemental Table 2: RRVs of various shRNA configurations targeting *IDO-1*, *PDL1* and *TGF-β2*

shRNA configuration	Target with Down-regulation Activity > 75% [1]			
	GFP*	IDO-1*	PDL1*	TGF-β2*
H1-shRNA	0/2	NA	NA	NA
U6-shRNA	0/2	0/4	0/4	0/4
U6-miRshRNA	1/2	2/4	2/4	2/4
H1-miRshRNA	NA	1/1	1/1	NA
RSV-miR shRNA	NA	1/1	1/1	NA
RSV-yCD2-miR shRNA	NA	0/1	0/1	NA
RSV-yCD2-U6-miR shRNA	NA	0/1	0/1	NA

1. Ratios of the number of sequences that showed > 75% down-regulation activity to the number of sequences tested.

* Matching siRNA sequences tested in different configurations for each targets

Supplemental Table 3: RRVs of various shRNA configurations targeting *PDL1*.

Vector Configuration	PD-L1		
	N	Down-regulation activity >50% [1]	Down-regulation activity >75% [2]
U6-shRNA *	4	3/4	0/4
U6-miR30shRNA *	4	4/4	2/4
H1-miR30shRNA *	1	1/1	1/1
RSV-miR30shRNA *	1	1/1	1/1
RSV-yCD2-miR30shRNA *	1	1/1	0/1
RSV-yCD2-U6-miR30shRNA *	1	0/1	0/1

1. Ratios of the number of sequences that showed > 50% down-regulation activity to the number of sequences tested.
2. Ratios of the number of sequences that showed > 75% down-regulation activity to the number of sequences tested.

* Matching siRNA sequences tested in different configurations for each targets

N = number of sequence tested

Supplemental Table 4: Antisense sequences targeting human *IDO-1*, *PDL-1* and *TGF-β2*

Target	Accession #/Clone ID	Antisense seq (5- to 3')
IDO-1	NM_002164	
Seq 1		TTCATTAAATCAGTGCCTCCA
Seq 2		TTTGGGTTCACATGATCGTGG
Seq 3		TGTTTAGCAATGAACATCCAG
Seq 4		TTACTTTGATTGCAGAAGCAG
PDL-1	NM_014143	
Seq 1		TTTCACATCCATCATTCTCCC
Seq 2		TTGATGGTCACTGCTTGTCCA
Seq 3		TTTCACAGTAATTCGCTTGTA
Seq 4		TCAATTGTCATATTGCTACCA
TGF-β2	NM_003238	
Seq 1		TTAGATGGTACAAAAGTGCAG
Seq 2		AACAGCATCAGTTACATCGAA
Seq 3		TTCACAACTTTGCTGTCGATG
Seq 4		TAGTTTTCTGATCACCACTGG

Supplemental Figure 1

A

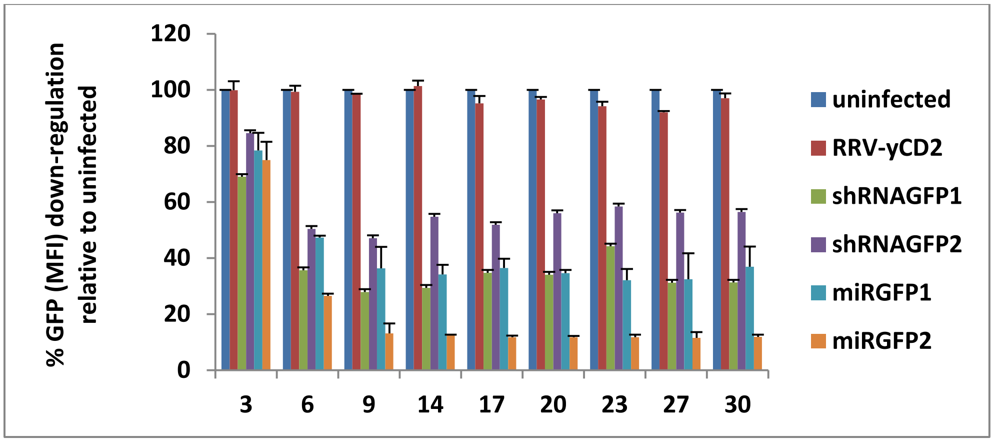

B

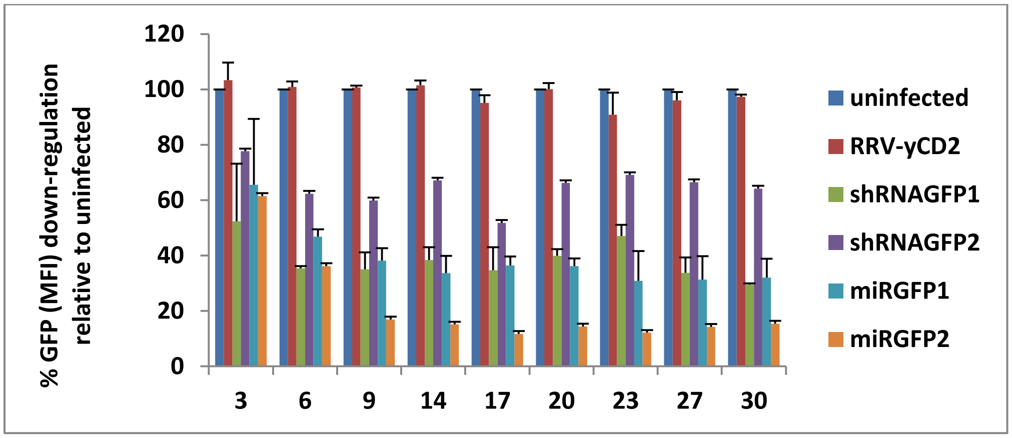

Figure S1: RRV-U6-miRGFP has higher GFP down-regulation activity than RRV-U6-shGFP. RRVs produced from transient transfection in 293T were used to infect U87-MG/GFP cells at MOI of 1 (A) or MOI of 10 (B). The GFP down-regulation activity was evaluated and quantified by flow cytometric analysis for mean fluorescent intensity (MFI). Both uninfected cells and cells infected with RRV-yCD2 were included as positive controls. The percentage of GFP down-regulation is relative to the uninfected cells. The data shown represent mean ± SD of triplicates performed from one of two independent experiments

Supplemental Figure 2

A

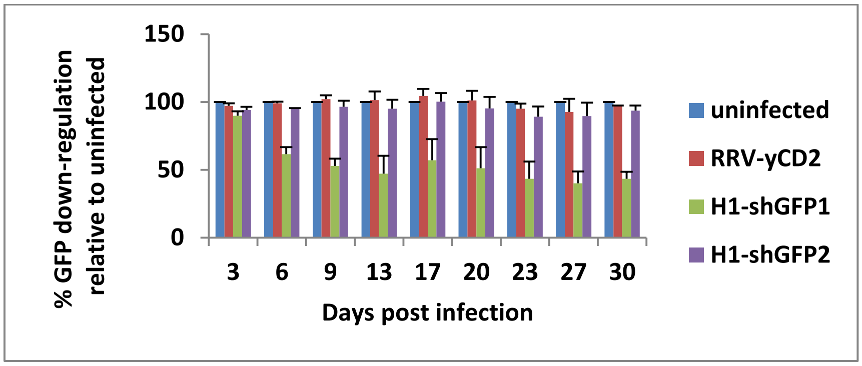

B

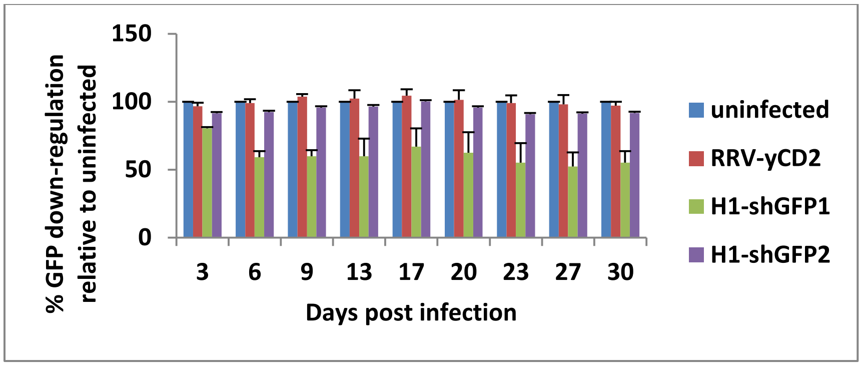

C

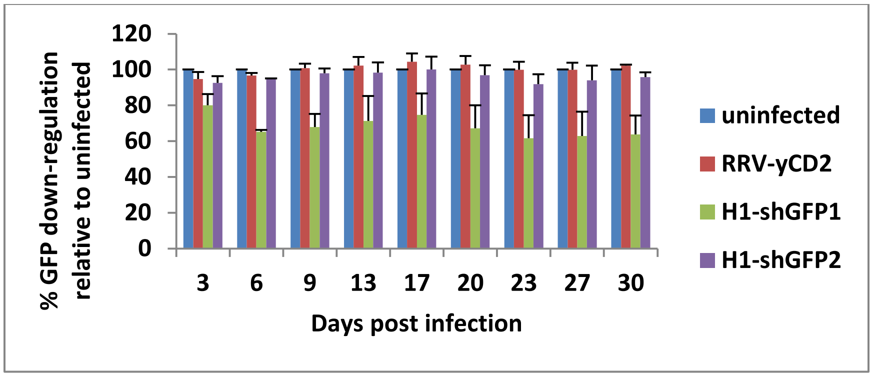

Figure S2: GFP down-regulation activity of RRV-H1-miRGFP RRVs produced from transient transfection in 293T were used to infect U87-MG/GFP cells at MOI of 0.1 (A), MOI of 1 (B) or MOI of 10 (C). The GFP down-regulation activity was evaluated and quantified by flow cytometric analysis for mean fluorescent intensity (MFI). Both uninfected cells and cells infected with RRV-yCD2 were included as positive controls. The percentage of GFP down-regulation is relative to the uninfected cells. The data shown represent mean ± SD of triplicates performed from one of two independent experiments.

Supplemental Figure 3

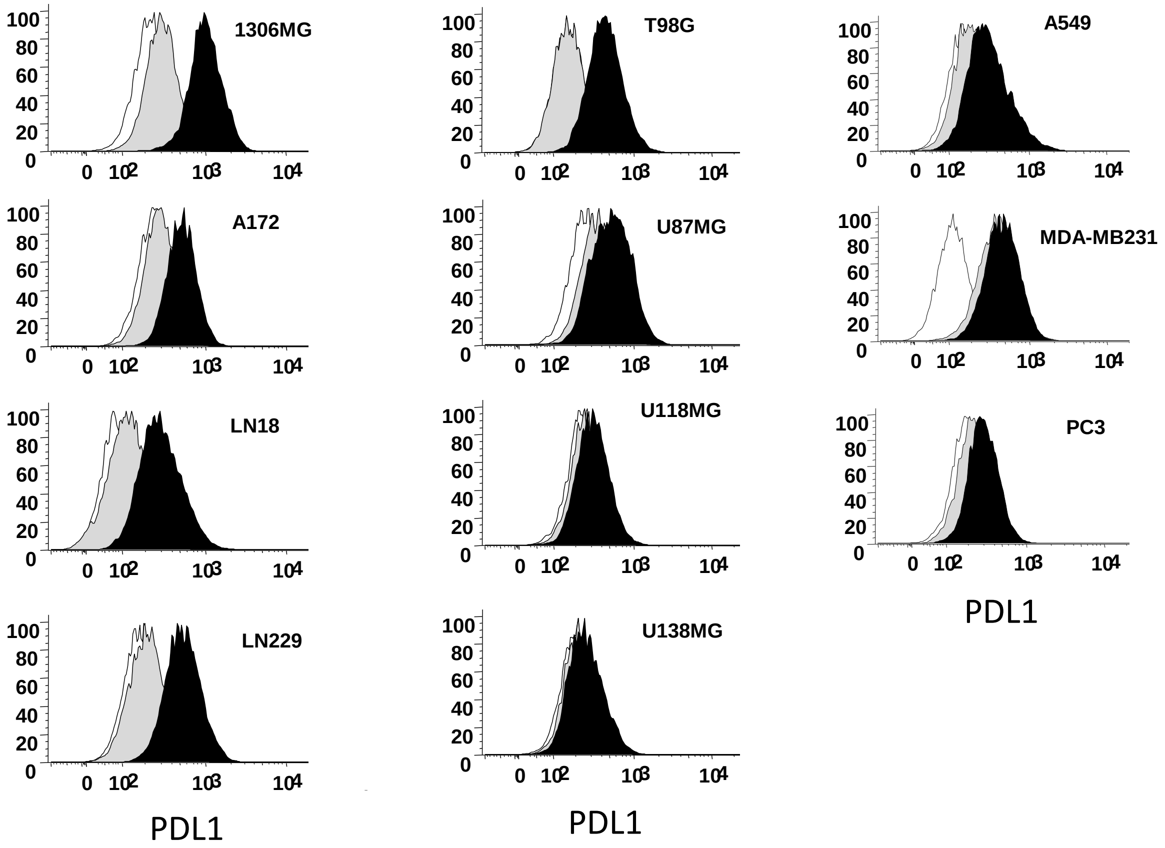

Figure S3: PDL1 cell surface expression in various tumor cell lines. Human glioblastoma cell lines 1306MG (a kind gift from Dr. Carol A Kruse), U87-MG, LN-18, LN-229, T98G, U118MG, U138MG, (ATCC), human lung carcinoma cells A549, human breast cancer cell line MDA-MB-231 and human prostate cancer cells PC-3 (ATCC) were cultured in complete culture medium containing 10% FBS (Hyclone), sodium pyruvate, GlutaMAX (Thermo Fisher Scientific), and antibiotics at penicillin 100 IU/mL, streptomycin 100 IU/mL (Corning). Cells treated with or without IFNγ at 500 IU/mL for 36 hours were harvested using cell dissociation buffer (10 mM EDTA/PBS) and washed with autoMACS rinsing solution containing 10% BS (Miltenybiotec, cat # 130-091-222 and cat # 130-091-376). Cell pellets were resuspended in 100 μL autoMACS rinsing solution with isotype control (eBioSciences, cat # 12-4714) or anti-human PDL1-PE-conjugated antibody (eBioSciences, cat # 12-5983) according to the manufacturer's protocol. Stained cells were washed with autoMACS rinsing solution and resuspended in 600 μL autoMACS rinsing solution for flow cytometric analysis. Isotype control (open histograms); basal PDL1 expression (gray filled histograms); and IFNγ-induced PDL1 expression (black filled histograms)

Supplemental Figure 4

A

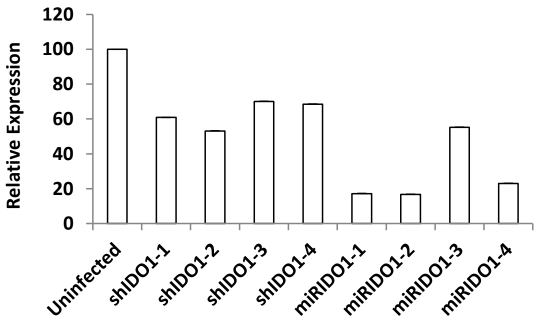

B

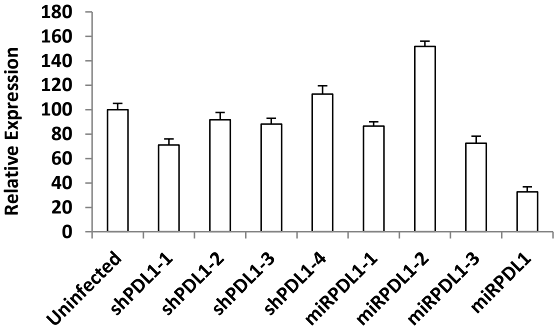

C

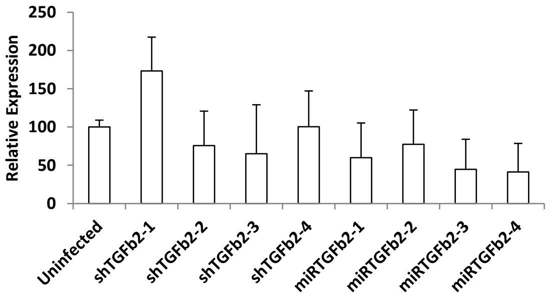

Figure S4: Quantification of *IDO-1*, *PDL1* and *TGF-β2* gene expression in LN-18 cells infected with RRVs expressing shRNA or miRshRNA. RNA was extracted from infected LN-18 cells using the RNeasy Kit with DNase I treatment (Qiagen). Reverse transcription was carried out with 100 ng total RNA using High Capacity cDNA Reverse Transcription Kit (ABI). Quantitative PCR (qPCR) was performed using TaqMan Universal PCR Master Mix, No AmpErase UNG (ABI, P/N # 4324018) and Taqman gene expression primers (ABI), (ID # Hs01125301_m1 for *PDL1*, ID# Hs 00984148_m1 for *IDO-1*, ID # Hs00234244_m1 for *TGF-β2*, and Hs99999905_m1 for *GAPDH*) according to manufacturer's protocol. Relative expression is calculated using the $2^{-\Delta\Delta(Ct)}$ method with respect to expression in uninfected cells and converted to percentage.

Supplemental Figure 5

A

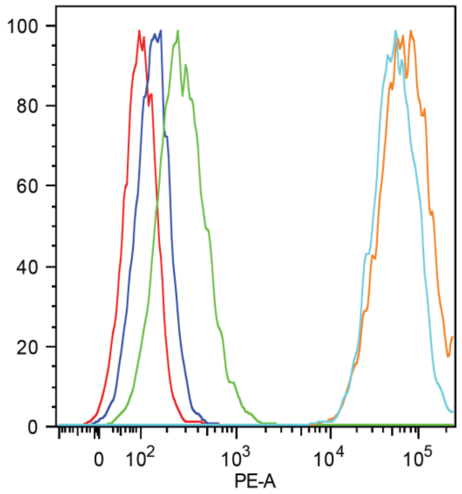

B

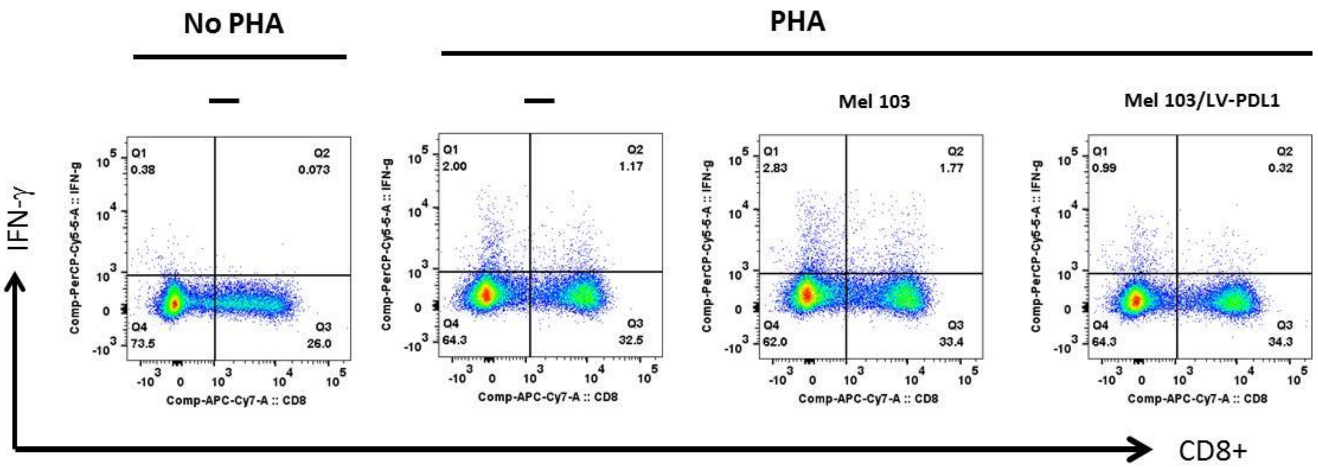

Figure S5: Cell surface expression of PDL1 in Mel 103 cells and Mel 103 cells overexpressing PDL1 (Mel103/LV-PDL1) in the presence or absence of IFNγ induction. Isotype control (red histogram); basal PDL1 expression (dark-blue histogram); IFNγ-induced PDL1 expression (green histogram) in Mel 103 cells. PDL1 overexpression (light-blue histogram) and IFNγ-induced (orange histogram) in Mel 103/LV-PDL1 cells. (B) PHA-induced PBMCs were cultured alone or cocultured with Mel 103 cells or Mel 103/LV-PDL1 cells. Intracellular IFNγ in CD8+ population with gating set based fluorescent minus one was analyzed by flow cytometry.

Supplemental Figure 6

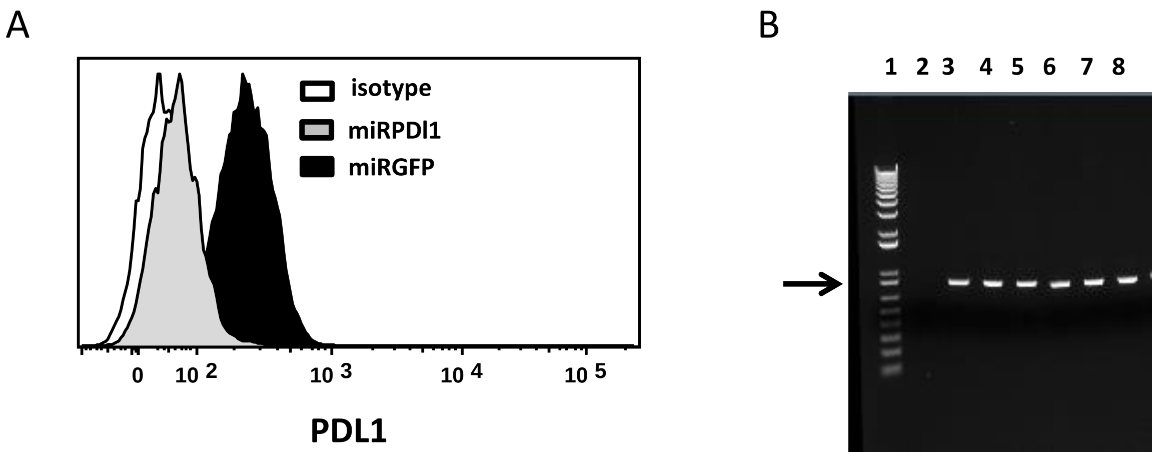

Figure S6: PDL1 down-regulation and vector stability of MDA-MB-231BR cells infected with RRV-miRPDL1. (A) MDA-MB-231BR cells infected with RRV-miRGFP and RRV-miRPDL1 were stained for PDL1 cell surface expression with PDL1 antibody and analyzed by flow cytometry. (B) Vector stability of RRV-miRGFP and RRV-U6-miRPDL1 infected MDA-MB-231BR cells was analyzed by end-point PCR 14 days post infection. Lane 1: DNA molecular marker (1kb plus marker, Invitrogen); lane 2, non-template control; lanes 3 and 6 are positive controls using the corresponding plasmid DNA as the templates; lane 4 (MOI of 0.1) and lane 5 (MOI of 1): RRV-miRGFP; lanes 7 (MOI of 0.1) and 8 (MOI of 1) : RRV-miRPDL1.
