# Supplementary material for: Retroviral Replicating Vector Delivery of miR-PDL1 Inhibits Immune Checkpoint PDL1 and Enhances Immune Responses In Vitro
Source: Mol Ther Nucleic Acids. 2016 Dec 10;6:221–32. doi: 10.1016/j.omtn.2016.11.007 (PMC5363416; doi:10.1016/j.omtn.2016.11.007)
Supplement: Document S2. Article plus Supplemental Information [file mmc2.pdf]

Molecular Therapy
Nucleic Acids
Original Article

Retroviral Replicating Vector Delivery of miR-PDL1 Inhibits Immune Checkpoint PDL1 and Enhances Immune Responses In Vitro

Amy H. Lin,[1,2] Christopher G. Twitty,[1,2,3] Ryan Burnett,[1] Andrew Hofacre,[1] Leah A. Mitchell,[1] Fernando Lopez Espinoza,[1] Harry E. Gruber,[1] and Douglas J. Jolly[1]

[1]Tocagen Inc., 3030 Bunker Hill Street, Suite 230, San Diego, CA 92109, USA

Tumor cells express a number of immunosuppressive molecules that can suppress anti-tumor immune responses. Efficient delivery of small interfering RNAs to treat a wide range of diseases including cancers remains a challenge. Retroviral replicating vectors (RRV) can be used to stably and selectively introduce genetic material into cancer cells. Here, we designed RRV to express shRNA (RRV-shPDL1) or microRNA30-derived shRNA (RRV-miRPDL1) using Pol II or Pol III promoters to downregulate PDL1 in human cancer cells. We also designed RRV expressing cytosine deaminase (yCD2) and miRPDL1 for potential combinatorial therapy. Among various configurations tested, we showed that RRV-miRPDL1 vectors with Pol II or Pol III promoter replicated efficiently and exhibited sustained downregulation of PDL1 protein expression by more than 75% in human cancer cell lines with high expression of PDL1. Immunologic effects of RRV-miRPDL1 were assessed by a trans-suppression lymphocyte assay. In vitro data showed downregulation of PDL1$^+$ tumor cells restored activation of CD8$^+$ T cells and bio-equivalency compared to anti-PDL1 antibody treatment. These results suggest RRV-miRPDL1 may be an alternative therapeutic approach to enhance anti-tumor immunity by overcoming PDL1-induced immune suppression from within cancer cells and this approach may also be applicable to other cancer targets.

INTRODUCTION

Tocagen has developed a retroviral replicating vector (RRV) system that allows preferential viral replication in tumor cells.[1] Toca 511 (vocimagene amiretrorepvec) is an investigational RRV that encodes a prodrug activating gene (optimized yeast cytosine deaminase, yCD2). Toca 511 infects and spreads in tumors. A prodrug 5-fluorocytosine (5-FC) is then administered leading to local production of high concentrations of the anti-cancer drug 5-FU only in infected cells. In clinical trials, 5-FC is administered as an investigational, extended-release tablet called Toca FC. This treatment regimen has shown favorable safety and tolerability profiles in addition to durable responses associated with shrinkage and stable disease by independent radiologic review of serial MRI brain scans in patients receiving higher doses of Toca 511 in phase 1 trials. Patients showed durable survival advantages compared to historical controls, especially in those with radiologic responses and stable disease.[2] Toca 511 in combination with Toca FC is currently under investigation in an international, multicenter, randomized phase 2/3 trial in patients with recurrent high grade glioma (NCT02414165).

RNAi via either microRNA (miRNA) or small interfering RNA (siRNA) is an evolutionarily conserved biological process utilizing endogenously processed double-stranded RNA of 21–25 nucleotides in length to inhibit gene expression. The guide strand of the processed siRNA is incorporated into an RNA induced silencing complex (RISC) and binds to the target mRNA. The formation of the RNA duplex results in the downregulation of gene expression, either by repression of protein translation or by degradation of mRNA, depending on the degree of mismatch between the RNA incorporated onto the RISC and its target mRNA. The silencing efficiency is known to be influenced greatly by the sequence as well as the extent of siRNA expressed intracellularly or delivered exogenously.[3,4] Although therapeutic applications of RNAi have been reported in both pre-clinical and clinical settings,[5–7] efficient delivery of synthetic siRNA molecules to various organ tissues or endogenous expression of shRNA in targeted organ tissues in vivo remains a barrier to implementation.[4,8]

The use of small hairpin RNA (shRNA) designed to express intracellularly was implemented as a tool to overcome some of the delivery issues that exogenous siRNA delivery methods encountered including duration of inhibition and induction of undesirable immune response.[3,9,10] With increasing understanding of miRNA processing, Zeng et al. first demonstrated that artificially designed miRNA derived from miRNA-30 (miRshRNA) can function as siRNA.[11,12] Several groups compared the biological process and function between shRNA versus miRshRNA and reported that miRshRNA

Received 11 August 2016; accepted 16 November 2016;
http://dx.doi.org/10.1016/j.omtn.2016.11.007.

[2]These authors contributed equally to this work.

[3]Present address: OncoSec Medical, 5820 Nancy Ridge Drive, San Diego, CA 92121, USA

Correspondence: Douglas J. Jolly, Tocagen Inc., 3030 Bunker Hill Street, Suite 230, San Diego, CA 92109, USA.
E-mail: djolly@tocagen.com

This is an open access article under the CC BY license (http://creativecommons.org/licenses/by/4.0/).

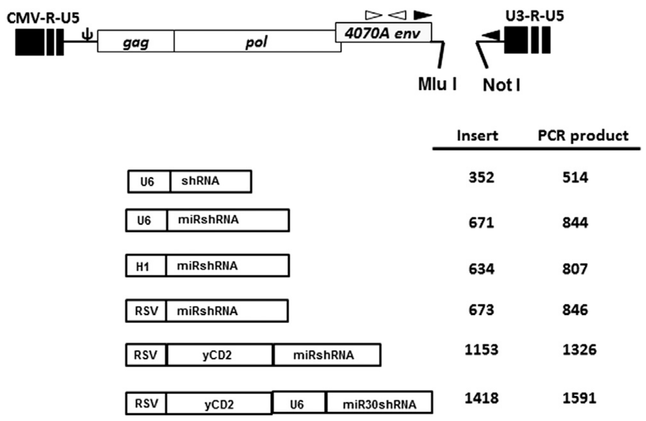

Figure 1. Schematic Diagram of Plasmid DNA of RRVs Expressing shRNA, miRshRNA, and yCD2-miRshRNA

shRNA, miRshRNA, and yCD2-miRshRNA in various configurations inserted downstream of the 3′ UTR at Mlu I and Not I restriction sites. The filled arrowheads indicate primer set (5–127 and 3–37) used for endpoint PCR to evaluate the integrity of insert in proviral DNA. The open arrowheads indicate primer set used for qRT-PCR to measure the viral genome expression in the supernatant of RRV infected cells. The numbers indicate the lengths in base pair of inserts or PCR products for each configuration.

are processed more efficiently than shRNA and have less cytotoxicity linked to saturation of endogenous RNAi machinery.[10,13–15] Since the development of the miRshRNA configuration, it has been widely incorporated into non-viral expression vectors as well viral vectors,[13,16–19] Schaser et al. have previously shown the feasibility of incorporating a single miRshRNA cassette into a MLV-based RRV and observed anti-tumor function in vivo.[18] However, the vector designed was limited to the commonly used U6 promoter miRshRNA cassette, and vector characterization was also limited in the report. Thus, the generality of this result remains untested. In this study, we investigated the possibility to incorporate different miRshRNA cassettes using different promoters. We also investigated the possibility to generate a combinatorial RRV that contains two therapeutic agents. We characterized the RRVs by assessing their vector stability, replication kinetics, and downregulation activity. Importantly, we showed in vitro that RRV expressing miRshRNA targeting *PDL1* effectively restored immune activation suppressed by PDL1+ tumor cells and demonstrated bio-equivalency compared to anti-PDL1 antibody treatment. Given the reported favorable safety profile of Toca 511,[2] immune modulation within the tumor microenvironment by cancer-selective RRV-miRPDL1 as opposed to systemic delivery of clinically approved anti-PD1 and anti-PDL1 antibodies may offer clinical potential alone or in combination with other therapeutic agents to help activate the immune system.

RESULTS

MicroRNA30-Based shRNA Expressed by RRV Has Higher Potent Gene Downregulation Activity than Conventional shRNA

MicroRNA30-based shRNA (miRshRNA) encoded in expression plasmid DNA was shown to have higher gene downregulation activity than conventional shRNA.[20,21] In this study, we first asked which miRshRNA expression configuration works best from RRV. We generated human U6 promoter driven-MLV-based RRV expressing conventional shRNA (RRV-shGFP) or microRNA30-based shRNA (RRV-miRGFP) targeted to GFP (Figure 1). We tested two sequences (shGFP1, shGFP2, miRGFP1, and miRGFP2) that have been reported to have at least 75% GFP downregulation activity.[18,22] RRVs were produced by transient transfection in 293T cells, and viral titer values were determined on PC3 cells by qPCR as previously described[23] (Table S1). The GFP downregulation activity was evaluated and quantified by flow cytometric analysis during a 30-day course of infection in U87-MG cells stably expressing high levels of GFP. Our data showed that at a MOI of 0.1, all four constructs were able to achieve at least 50% downregulation activity as early as day 6 post infection. However, >75% GFP downregulation activity was observed only with RRV-miRGFP2, and the downregulation activity persisted up to 30 days in culture (Figure 2A). GFP downregulation activity in the higher MOI did not further improve when cells were infected with a MOI of 1 or 10 (Figure S1), suggesting that the amount of siRNA produced in cells initially infected with a MOI of 0.1 was sufficient to downregulate GFP expression. Although RRV-shGFP1 appears to be more potent than RRV-shGFP2, and the difference in downregulation activity observed between the two vectors was further confirmed using a human H1 promoter (Figure S2), opposite trending was observed between RRV-miRGFP1 and RRV-miRGFP2 (Figure 2A). Also, in contrast to RRV-shGFP1 and RRV-U6-miRGFP1, which showed similar GFP downregulation activity, a significant difference in downregulation activity was observed between RRV-shGFP2 and RRV-miRGFP2. To ensure that the difference in downregulation activity between the two vectors was not due to emergence of a deletion mutant during viral replication, end-point PCR using primers spanning the U6 promoter-shGFP and U6 promoter-miRGFP cassette of viral genome was performed from genomic DNA of infected cells 30 days post infection (Figure 2B). Together, the data suggest that the miRshRNA configuration appears to be more potent than conventional shRNA.

To further extend the observation that miRshRNA configuration has better downregulation activity than shRNA configuration of the matching sequence in RRV, we generated additional four matching pairs of siRNA sequences in shRNA and miRshRNA configurations targeted to human *IDO-1*, *PDL1*, and *TGF-β2*, key factors known to play an immune suppressive role in the tumor microenvironment. For these targets, the human glioma LN-18 cell line was used to evaluate downregulation activity due to its high expression level of IDO-1, PDL1, and TGF-β2 with or without induction by IFNγ (Figure S3). Similar to the observation made with RRV-shGFP and RRV-miRGFP vectors bearing the U6 promoter, the miRshRNA configuration consistently has higher downregulation activity than the shRNA configuration in all four targets tested (Figure S4; Tables S2 and S3).

miRshRNA Expressed by RRV Does Not Trigger Type I Interferon Response

We have previously shown that in vitro RRV does not trigger type I interferon (IFN) response.[24] However, type I IFN response induced

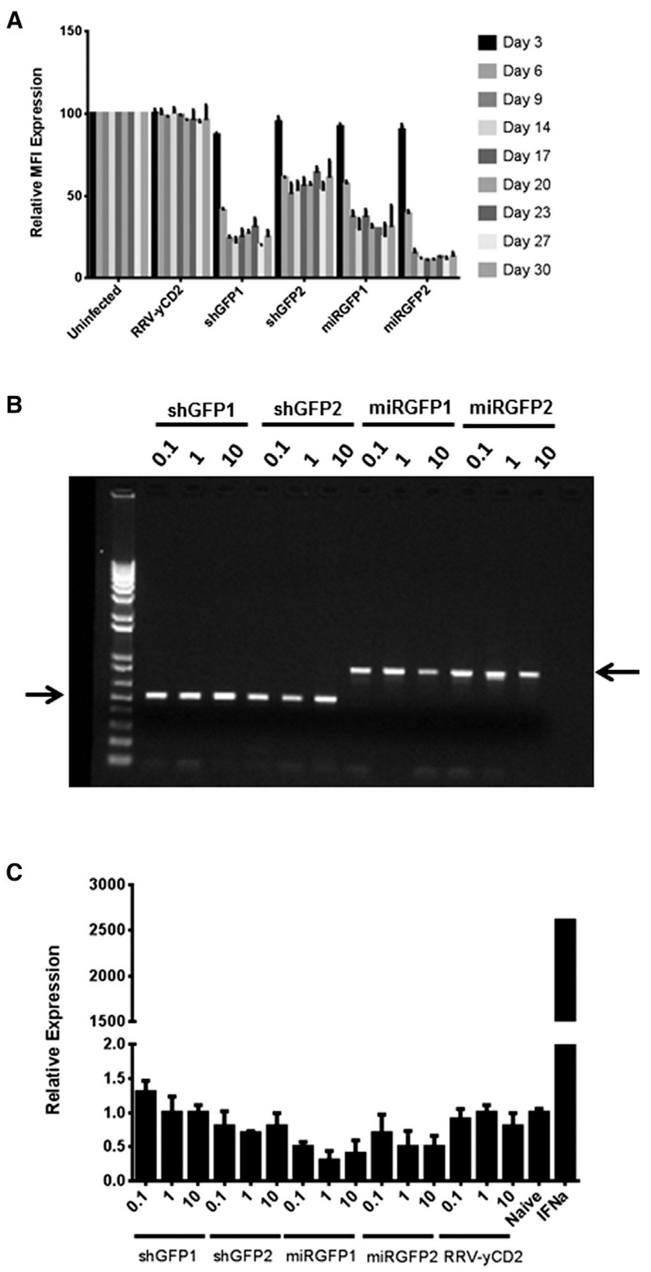

Figure 2. RRV-U6-miRGFP Has Higher GFP Downregulation Activity than RRV-U6-shGFP and Does Not Trigger IFN Response

(A) RRVs produced from transient transfection in 293T were used to infect U87-MG/GFP cells at a MOI of 0.1 (see Figure S1 for MOI of 1 and 10). The GFP downregulation activity was evaluated and quantified by flow cytometric analysis for MFI. Both uninfected cells and cells infected with RRV-yC2 were included as a positive control. The percentage of GFP downregulation is relative to the uninfected cells. The data shown represent mean ± SD of triplicates performed from one of multiple independent experiments. (B) Vector stability of RRV-U6-shGFP and RRV-U6-miRGFP using genomic DNA from infected U87-MG/GFP cells at 30 days post infection was analyzed by endpoint PCR. The DNA molecular marker (1 Kb Plus marker, Invitrogen) is included in the first lane. The right and left arrows indicate the expected size of the PCR products for the RRV-shGFP (514 bp) and for the RRV-miRGFP (844 bp), respectively. (C) Induction of *OAS1* gene expression induced by IFN response in U87-MG cells infected with RRV-U6-shGFP or RRV-U6-miRGFP at a MOI of 0.1, 1, and 10. U87-MG cells treated with exogenous recombinant human IFNα were included as positive control. The values are presented as the means ± SD of triplicates.

by synthetic siRNA has been reported and also in some cases in which siRNA are processed intracellularly from shRNA.[10,25] One of the advantages of generating siRNA from the miR30-derived shRNA backbone is potential further minimization of type I IFN response.[9,10] To determine whether miRshRNA expressed by RRV triggers type I IFN response, we used U87-MG cells which have previously been shown to be functional in producing type I IFN and responsive to type I IFN.[24] In this experiment, we infected U87-MG cells at a MOI of 0.1, 1, and 10 and obtained the cellular RNA 16 hr post infection. We then measured the gene expression of 2′5′-oligoadenylate synthetase 1 (*OAS1*), which is commonly used as an indicator for cellular type I IFN response. Figure 2C showed that U87-MG cells treated with exogenous IFNα induced OAS1 gene expression by more than 25,000-fold compared to naive U87-MG cells. As previously reported,[24] U87-MG cells infected with RRV-yCD2 (aka Toca 511) did not induce an IFN response that leads to robust induction of *OAS1* expression as indicated by minimal change of OAS1 gene expression within 2-fold. Likewise, neither RRV-shGFP nor RRV-miRGFP induced IFN responses in U87-MG cells (Figure 2C). In addition, we have indirectly shown that RRVs containing various miRshRNA sequences targeted to *IDO-1* and *PDL1* or *TGF-β2* also did not trigger IFN response in U87-MG cells nor in LN-18 cells as indicated by their viral replication kinetics (data not shown).

RRV-miRPDL1 Replicates Efficiently in LN-18 Cells and Has Potent Downregulation Activity against PDL1

After confirming that the miRshRNA configuration has higher downregulation activity than shRNA configuration using several targets, we chose one of RRV-miRPDL1 vectors that exhibited potent PDL1 downregulation activity screened in LN-18 cells. We first demonstrated that the replication kinetics of RRV-miRPDL1 is slightly delayed, but not statistically significant ($p = 0.1915$) compared to that of RRV-yCD2 in LN-18 cells (Figure 3A). The viral replication kinetics also allowed us to determine the time needed for virus to reach maximally infectivity for evaluating gene downregulation activity. Figure 3B shows that infection of RRV-miRPDL1 in LN-18, of which PDL1 expression was further induced by exogenous IFNγ, potently downregulated PDL1 gene expression. PDL1 downregulation activity at the protein level by RRV-miRPDL1 was also measured and quantified by flow cytometric analysis. Our results showed that RRV-miRPDL1 not only effectively downregulated *PDL1* RNA expression, it also effectively downregulated PDL1 protein expression by more than 75% (Figures 3C and 3D). In addition, our data showed that RRV-miRPDL1 genome remained stable in LN-18 cells over time, as no detectable deletion mutant was observed at day 30 post infection (Figure 3E). Altogether, our data indicate that RRV-miRPDL1 bearing the U6 promoter displays similar functional viral

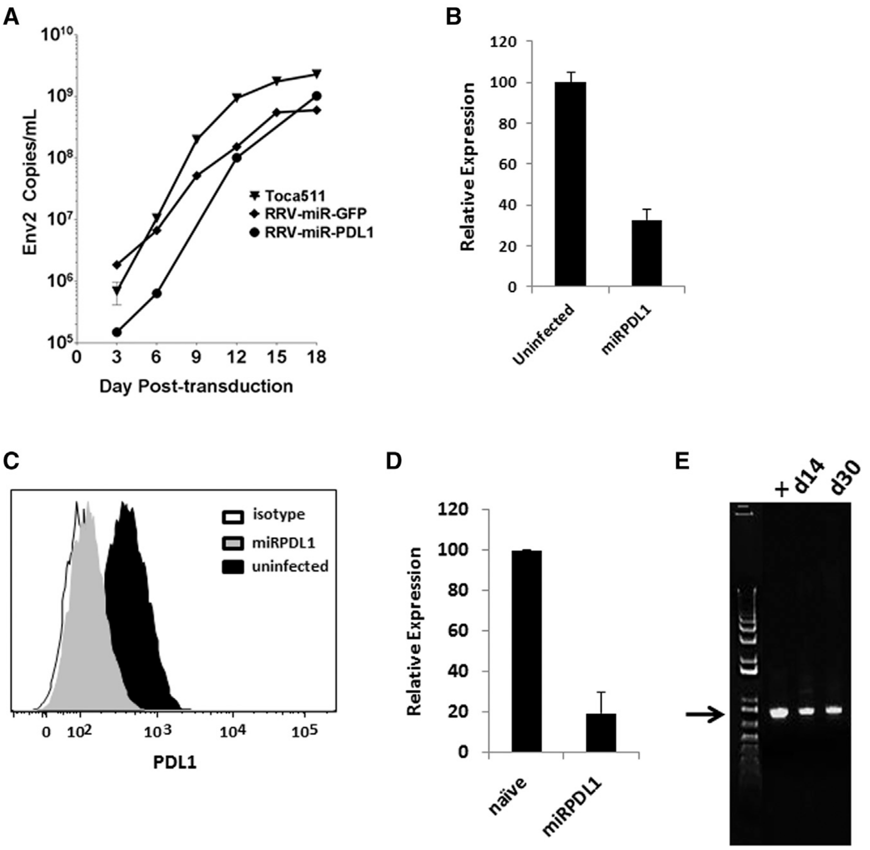

Figure 3. Vector Characterization of RRVs Expressing miRshRNA

(A) Replication kinetics of RRV-miRGFP and RRV-miRPDL1. The viral genome in the supernatants of infected LN-18 cells (MOI of 0.1) at indicated time points were quantified by qRT-PCR using primer set targeted to the env region (Figure 1). RRV-yCD2 was included as a positive control. A paired t test was performed and showed no statistically significant difference in replication kinetics between RRV-miRGFP versus Toca 511 ($p = 0.0778$) and between RRV-miRPDL1 versus Toca 511 ($p = 0.1915$). (B) *PDL1* gene expression in RRV-miRPDL1 infected LN-18 cells relative to uninfected cells. The values presented are means ± SD of triplicates. (C) LN-18 cells infected with RRV-miRPDL1 were stained for PDL1 cell surface expression with PDL1 antibody and analyzed by flow cytometry. The histogram shown represents data from one of the three independent experiments. (D) PDL1 cell surface expression in RRV-miRPDL1 infected LN-18 cells relative to uninfected cells. The values presented are means ± SD of three independent experiments. (E) Vector stability of RRV-miRPDL1 in LN-18 cells was analyzed by endpoint PCR at 14 and 30 days post infection. The DNA molecular marker (1 Kb Plus marker, Invitrogen) is included in the first lane. positive control using plasmid DNA as the template, +. The arrow indicates the expected size of the PCR products (844 bp).

characteristics (replication kinetics, titer values, and vector stability) as RRV-yCD2, and that it produces functional siRNA that can effectively downregulate targets of interest.

RRV-H1-miRPDL1 and RRV-RSV-miRPDL1 Show Equivalent PDL1 Downregulation Activity as RRV-miRPDL1

Endogenous miRNAs are normally transcribed by RNA polymerase II. Design of a Pol II promoter-miRNA configuration has been incorporated in some commercially available miRNA expression vectors presumably to mimic natural miRNA expression or to facilitate tissue-specific expression. We therefore replaced the human U6 promoter in RRV-miRPDL1 with a Pol II promoter from Rous sarcoma virus (RSV). In addition, we also generated an RRV-H1-miRPDL1 utilizing the human Pol III H1 promoter. As expected, the titer produced from RRV-H1-miRPDL1 and RRV-RSV-miRPDL1 were comparable to RRV-miRPDL1 (Table S1), and the viral replication kinetics among the three vectors were similar in LN-18 cells (Figure 4A) with no detectable deletion mutants observed during the course of viral replication (Figure 4B). Most importantly, PDL1 downregulation activity among the three vectors was also comparable in LN-18 cells (Figure 4C). Together, the data indicate that miRNA-based shRNA with a Pol II or Pol III promoter can be incorporated into an RRV genome and be efficiently expressed and processed to engage the RNAi pathway.

RSV-yCD2miRPDL1 and RSV-yCD2-U6-miRPDL1 Show Comparable yCD2 Protein Expression as RSV-yCD2, but Less PDL1 Downregulation Activity than U6-miRPDL1 and RSV-miRPDL1

Our recent data indicate that the mechanism of action of Toca 511 in combination with 5-FC is the cytotoxic effect of 5-FU on both tumor cells and tumor-associated immunosuppressive myeloid cells, resulting in potentiation of immune-modulated anti-tumor effects in a syngeneic mouse model (Mitchell et al., 2016, unpublished data). To further extend the immune-modulated therapeutic effect of Toca 511, we sought to express both yCD2 and miRPDL1 in RRV as a combinatorial therapy. Due to size limitation in the MLV viral genome,[26] and given that the RSV-miRPDL1 has equivalent downregulation activity as U6-miRPDL1, the IRES-yCD2 cassette in Toca 511 in which the yCD2 protein expression is mediated, an internal ribosomal entry site (IRES) was replaced with a RSV-yCD2 cassette in order to provide space to incorporate the miRPDL1 sequence into the viral genome. In this construct (RSV-yCD2-miRPDL1), the size of the cassette is approximately 1.1 kb, and the construct is designed so that the RSV promoter drives the expression of both the yCD2 gene and the miRPDL1 as a single transcript which mimics the way endogenous miRNAs are arranged in the host genome. In this configuration, the transcript pool, theoretically, is partitioned into two sub-pools. One sub-pool presumably stays in the nucleus and undergoes

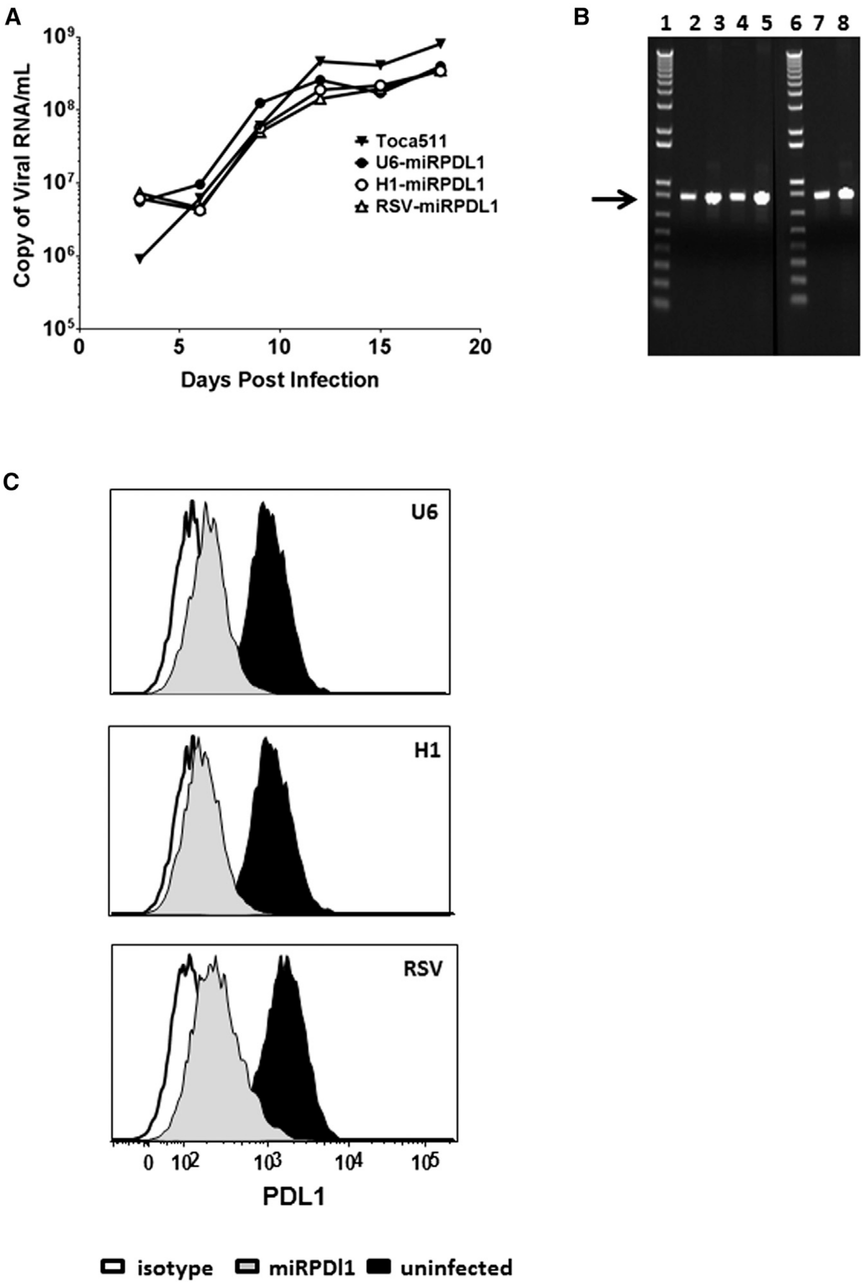

Figure 4. RRV-RSV-miRPDL1 and RRV-H1-miRPDL1 Exhibit Comparable PDL1 Downregulation Activity as RRV-miRPDL1

(A) Replication kinetics of RRV-RSV-miRPDL1 and RRV-H1-miRPDL1. The viral genome in the supernatants of infected LN-18 cells (MOI of 0.1) at indicated time points were quantified by qRT-PCR using primer set targeted to the *env* region (Figure 1). RRV-yCD2 and RRV-miRPDL1 (indicated as U6-miRPDL1 in the graph) were included as positive controls. (B) Vector stability of RRV-RSV-miRPDL1 and RRV-H1-miRPDL1 in LN-18 cells was analyzed by endpoint PCR at 14 and 30 days post infection. Lanes 1 and 6: DNA molecular marker (1 Kb Plus marker, Invitrogen); lanes 2, 4, and 7 are positive controls using the corresponding plasmid DNA as the templates; lane 3: RRV-H1-miRPDL1; lane 5: RRV-RSV-miRPDL1; and lane 8: RRV-miRPDL1. The arrow indicates the expected size of the PCR products. (C) LN-18 cells infected with RRV-miRPDL1 (U6), RRV-H1-miRPDL1 (H1), and RRV-RSV-miRPDL1 (RSV) were stained for PDL1 cell surface expression with PDL1 antibody and analyzed by flow cytometry.

miRNA processing, and another sub-pool is exported to the cytoplasm for yCD2 protein translation. In parallel, we also generated an additional RRV, which utilizes separate promoters to drive yCD2 and miRPDL1 expression. The size of the RSV-yCD2-U6-miRPDL1 cassette is approximately 1.4 kb, approaching the upper limit that the MLV viral genome can tolerate. In this configuration, yCD2 gene expression is driven by the RSV promoter, while the miRPDL1 is driven by the U6 promoter. We compared the two constructs to that of the RSV-yCD2 and RSV-miRPDL1 for their yCD2 protein expression and PDL1 downregulation activity, respectively.

Our data showed that viral replication kinetics of RRV-RSV-yCD2miRPDL1 and RRV-RSV-yCD2-U6-miRPDL1 in LN-18 cells are slightly delayed, but not statistically significant compared to that of RRV-RSV-yCD2 (p = 0.0649 and p = 0.0801, respectively) (Figure 5A). Unsurprisingly, a deletion mutant was readily detectable with RRV-RSV-yCD2-U6-miRPDL1 in LN-18 cells at day 14 post infection (Figure 5B). Noticeably, a similar result was observed in another combinatorial construct, RRV-RSV-yCD2-U6-miRIDO1 (data not shown), suggesting the genome instability observed is likely due to the size of the insert. The yCD2 protein expression level and PDL1 downregulation activity of the two constructs were also evaluated in maximally infected LN-18 cells. As might be expected, the yCD2 protein expression of RRV-RSV-yCD2 is less than that of RRV-yCD2, as the yCD2 protein translation mediated by IRES can occur from both unspliced and spliced viral genomes (Figure 5C). Also as expected, yCD2 protein expression levels in both RRV-RSV-yCD2-miRPDL1 and RRV-RSV-yCD2-U6-miRPDL1 is further decreased compared to that of RRV-RSV-yCD2 (Figure 5C). Furthermore, the PDL1 downregulation activity was markedly compromised in both constructs compared to that of RRV-miRPDL1 and RRV-RSV-miRPDL1 (Figure 5D). Together, the data suggest that although it is feasible to generate RRV to express two separate therapeutic entities from one promoter without compromising its viral genome integrity, therapeutic protein and miRshRNA expression may be reduced in such configurations.

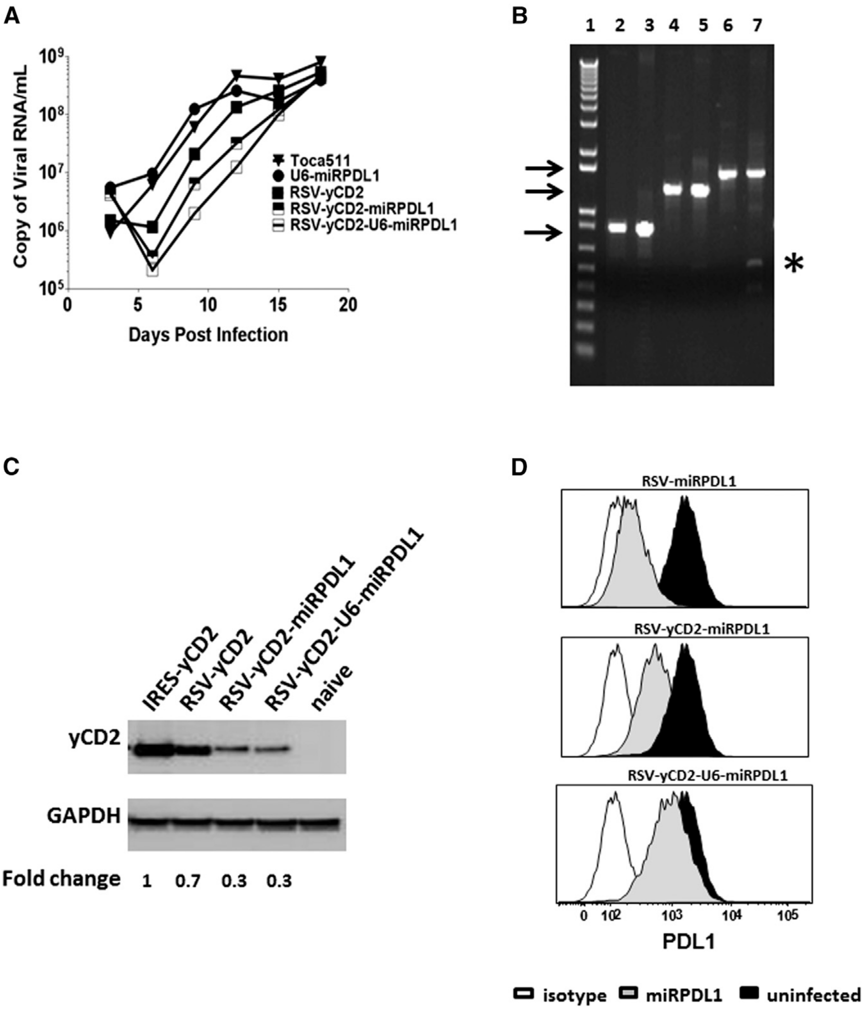

Figure 5. RRV-RSV-yCD2-miRPDL1 and RRV-RSV-yCD2-U6-miRPDL1 Express yCD2 Protein and Exhibit PDL1 Downregulation Activity

(A) Replication kinetics of RRV-RSV-yCD2-miRPDL1 and RRV-RSV-yCD2-U6-miRPDL1. The viral genome in the supernatants of infected LN-18 cells (MOI of 0.1) at indicated time points were quantified by qRT-PCR using primer set targeted to the *env* region (Figure 1). A paired t test was performed and showed no statistically significant difference in replication kinetics between RRV-RSV-yCD2-miRPDL1 versus RRV-RSV-yCD2 ($p = 0.0649$) and RRV-RSV-yCD2-U6-miRPDL1 ($p = 0.0801$). RRV-yCD2, RRV-RSV-yCD2, and RRV-miRPDL1 (indicated as U6-miRPDL1 in the graph) were included as positive controls. (B) Vector stability of RRV-RSV-yCD2-miRPDL1 and RRV-RSV-yCD2-U6-miRPDL1 in LN-18 cells was analyzed by endpoint PCR at 14 days post infection. Lane 1: DNA molecular marker (1 Kb Plus marker, Invitrogen); lanes 2, 4, and 6 are positive controls using the corresponding plasmid DNA as the templates; lane 3: RRV-RSV-miRPDL1; lane 5: RRV-RSV-yCD2-miRPDL1; and lane 7: RRV-RSV-yCD2-U6-miRPDL1. The arrows indicate the expected size of the PCR products (844 bp for RRV-RSV-miRPDL1; 1,326 bp for RRV-RSV-yCD2-miRPDL1; and 1,591 bp for RRV-RSV-yCD2-U6-miRPDL1). (C) yCD2 protein expression in LN-18 cell infected with RRV-yCD2, RRV-RSV-yCD2, RRV-RSV-yCD2-miRPDL1, RRV-RSV-yCD2-U6-miRPDL1, and naive cells. GAPDH is included as loading control. The numbers shown on the bottom of the immunoblot indicate fold change of yCD2 protein expression relative to RRV-yCD2. (D) LN-18 cells infected with RRV-RSV-miRPDL1, RRV-RSV-yCD2-miRPDL1, and RRV-RSV-yCD2-U6-miRPDL1 were stained for PDL1 cell surface expression with PDL1 antibody and analyzed by flow cytometry.

RRV-miRPDL1 Restores PHA-Stimulated T Cell Activation and Shows Equivalence of PDL1 Blocking Antibody In Vitro

The notion that the PD1:PDL1 axis contributes to immune suppression in the tumor microenvironment has been established in both animal models and in cancer patients.[27–29] Antibodies that disrupt either PD1 or PDL1 have had tremendous impact in patient outcomes, demonstrating the robust anti-tumor immune effects of these checkpoint inhibitors in an in vivo setting. Haile et al. described an in vitro co-culture system whereby suppression of PHA- stimulated PBMCs by allogeneic tumors could be reversed in a PDL1-dependent fashion with CD80-Fc fusion protein or with an anti-PD1 or anti-PDL1 antibody.[30] To determine if RRV-miRPDL1-mediated downregulation of PDL1 on tumor cells could alleviate T cell suppression in practice, we performed a similar PDL1-mediated trans-suppression co-culture experiment. Here, we evaluated if modulation of PDL1 expression on various tumor cell lines could alter PHA-stimulated activation of healthy donor PBMC as measured by intracellular expression of IFNγ or release of IFNγ into the supernatant. We first set up a co-culture system using the melanoma cell line Mel 103, which has low PDL1 cell surface expression independent of exogenous IFNγ treatment, as well as the same cell line with stable overexpression of PDL1 (Figure S5A). In this co-culture setting, secreted IFNγ measured by ELISA and intracellular IFNγ expression measured by intracellular cytokine staining were used to measure PBMC activation. When co-cultured with Mel 103 cells, IFNγ release by PHA-stimulated PBMC was only partially suppressed compared to PHA-stimulated PBMC alone, while intracellular IFNγ production remained unperturbed (Figure 6A). In contrast, when PHA-stimulated PBMCs were co-cultured with Mel 103 cells stably expressing high levels of PDL1, a marked reduction in the frequency of CD8+ T cells producing IFNγ, as well as bulk IFNγ release by PBMC was observed, suggesting a robust PDL1-mediated immune-suppressive effect (Figures 6A and S5B). We next developed another trans-suppression co-culture system using the human glioma tumor cell line LN-18 cells whose PDL1 cell surface expression can be upregulated by exogenous IFNγ treatment. In this experiment, uninfected or RRV-miRPDL1 infected LN-18 cells were pre-treated with IFNγ to upregulate their PDL1 cell surface expression prior to co-culturing

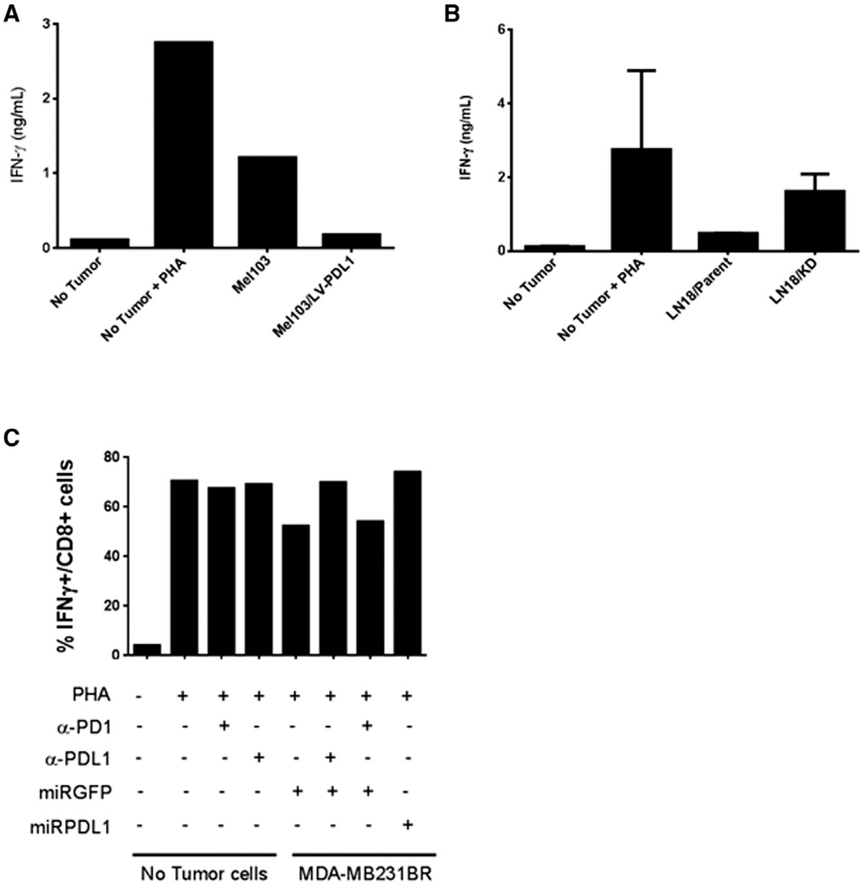

Figure 6. Downregulation of PDL1 by RRV-miRPDL1 Restores PHA-Stimulated T Cell Activation In Vitro

(A) PHA-induced PBMCs were cultured alone or co-cultured with Mel 103 cells or Mel 103 cells overexpression PDL1 (Mel 103/LV-PDL1). IFNγ production in the supernatant was measured by ELISA. (B) PHA-induced PBMCs were cultured alone or co-cultured with LN-18 parent cells or LN18 cells infected with RRV-miRPDL1 (LN18/KD). IFNγ production in the supernatant was measured by ELISA. (C) PHA-stimulated PBMCs were cultured alone or co-cultured with RRV-miRGFP infected MDA-MB-231BR cells in the presence or absence of anti-PDL1 and anti-PD1 antibody and compared to that co-cultured with RRV-miRPDL1 infected MDA-MB-231BR cells. The intracellular IFNγ expression in the $CD3^+/CD8^+$ gated population was analyzed by flow cytometry. The values present percentage of $IFN\gamma^+$ cells from one of multiple experiments.

with PHA-stimulated PBMCs. Consistent with the data observed using Mel 103 and Mel 103/LV-PDL1 cells, co-culture of LN-18 cells pre-treated with IFNγ and expressing high levels of PDL1 suppressed PHA-stimulated PBMC activation. In contrast, downregulation of PDL1 by RRV-miRPDL1 in LN-18 cells restored immune activation as indicated by an increase in IFNγ production (Figure 6B).

To eliminate the potential pleiotropic effects of IFNγ pre-treatment in the trans-suppression co-culture assay, we used the human breast cancer cell line MDA-MB-231BR, which has a high PDL1 basal cell surface expression level (Figure S6A). Consistent with data from LN-18 cells, RRV-miRPDL1 stably downregulates PDL1 cell surface expression in MDA-MB-231BR for at least 30 days after infection (Figure S6A). Infection of MDA-MB-231BR with RRV-miRGFP, a control RRV expressing a non-PDL1 target, does not affect PDL1 cell surface expression and, importantly, does not alleviate the tumor-dependent, PDL1-mediated immune suppression of IFNγ+ $CD8^+$ T cells. To confirm the necessity of PDL1 engagement in this assay, anti-PD1 and anti-PDL1 blocking antibodies were also included. $PDL1^+$ tumor cells (MDA-MB-231BR cells infected with RRV-miRGFP) in the presence of an anti-PDL1 blocking antibody were unable to suppress CD8+ T cell activation as indicated by the increased frequency of IFNγ+/CD8+ T cells (Figure 6C). Similarly, downregulation of PDL1 in MDA-MB-231BR cells by RRV-miRPDL1 equally restored $CD8^+$ T cell activation. While an anti-PD1 blocking antibody did not effectively restore CD8+ cell activation (likely due to an insufficient amount of blocking antibody), disruption of the PDL1:PD1 axis on tumor cells and lymphocytes either by RRV-miRPDL1-mediated downregulation or an anti-PDL1 blocking antibody provides evidence for a substantial immunological benefit from RRV-miRPDL1.

DISCUSSION

Effective delivery and persistence of RNAi-based products in target tissues has been an ongoing challenge for the use of RNAi-based therapeutic and research strategies. RRV have some properties, such as cell entry efficiency, tumor specificity, expected persistence, and a growing safety and drug activity record in humans that suggest that these delivery vectors could allow effective use of RNAi strategies. However, this potential utility has not been fully investigated to date. Among the unknowns are the RRV configurations that will allow significant reliable downregulation of targeted genes. We reasoned that this locally active modification of the tumor microenvironment presented opportunities to amplify pre-existing or nascent anti-tumor immune responses in environments that monoclonal antibodies cannot penetrate, and/or without the risk of systemic side effects, as have been seen with "checkpoint inhibitor" strategies with systemic administration of monoclonal antibodies against CTLA4, PD1/PDL1, or combinations thereof.[31,32]

We present here a comparison of multiple configurations of RRVs that allow expression of shRNA or miRshRNA and subsequent

processing to siRNA to optimize downregulation of key molecules involved in immune-suppression in the tumor microenvironment. In addition, we demonstrated the feasibility of using a Pol II promoter to drive expression of both coding and non-coding sequences with a single RRV vector, opening up the possibility of combining different forms of stimulation of anti-tumor immunity with RRV. We have further shown the potential functional utility of the levels of PDL1 downregulation achieved by an RRV expressing miRshRNA in PDL1$^+$ tumor cells by restoration of immune activation in an in vitro co-culture setting. This immune restoration demonstrates the potential for functional activity of local inhibition of immune checkpoint activity in the tumor microenvironment. Furthermore, the level of restoration is shown to be similar to that achieved with an anti-PDL1 antibody. However, it is important to point out that such an in vitro system does not exactly mimic the tumor microenvironment in which infiltrating T lymphocytes present are antigen specific. In addition, in the allogeneic settings both in vitro and in vivo, the restoration of immune response varies among tumor cell lines, as well as PBMC from allogeneic donors presumably due to the level of HLA mismatch.[33] Employment of antigen-specific tumor-infiltrating lymphocytes, syngeneic mouse model, or humanized mouse model system would be useful to support potential utilities for clinical use.

We have previously shown that RRV alone does not lead to type I IFN production from infected cells, and RRV particles can actively suppress type I IFN production.[24] This is different from most viral vectors including lentiviral vectors.[34,35] However, to rule out the possibility that persistent overexpression of miRshRNA with a 21-nucleotide fully complementary passenger strand could trigger a type I IFN response,[36,37] we have demonstrated that neither shRNA nor miRshRNA expressed intracellularly triggers a type I IFN response when incorporated into the RRV genome. This observation is further extended by showing that RRVs containing miRshRNA of sequences targeted to *IDO-1*, *PDL1*, or *TGF-β2* have similar viral replication kinetics as Toca 511 (data not shown). Therefore, IFN-meditated inhibition of delivery and subsequent spread of RRV expressing miRshRNA seems unlikely to be an issue in research and clinical settings.

A further potential issue is that non-coding RNA sequences such as shRNA and miRNA in a retrovirus genome could introduce encrypted splice sites that cannot be removed for functional reasons. For example, processing and stability of miRNA30 could be affected by such changes. Therefore, we compared the vector stability of the RRV-H1-miRPDL1, RRV-RSV-miRPDL1, and RRV-miRPDL1 bearing different internal promoters. Our short-term vector stability data indicate that miRNA30-derived miRshRNA in general is stable in the RRV genome in various cell lines tested.

Another theoretical concern for RRV expressing non-coding RNA sequences, which undergoes RNA processing, is the potential interference of viral RNA processing. Although the shRNA and miRshRNA sequences in our design were expressed by an internal promoter, the viral genome transcribed by the viral LTR promoter presumably includes the shRNA and miRshRNA sequences. Although no significant differences in viral titer were observed in virus produced from transiently transfected cells, miRNA processing could potentially compromise the viral genome processing, viral particles production, and thus delay viral spread. However, data from replication kinetics of the three RRV-miRPDL1 configurations (H1, U6, and RSV) in LN-18 cells suggest that that viral production was not significantly compromised when miRshRNA are incorporated as part of the viral genome. It has been suggested that dimerization of the MLV RNA viral genomes could occur as early as during transcription.[38] Thus, it is possible that the dimerization could prevent the initial processing of miRNA by Drosha in the nucleus. It is also possible that the formation of the secondary structure of miRNA could be prevented by the extensive base-pairing of the viral RNA genome sequence and, therefore, not amenable to miRNA processing by Drosha. The lack of impact on viral replication suggests that the majority of processing of shRNA and particularly the miRshRNA probably originated from the transcript mediated by the internal promoter. In addition, we also showed that there is no significant difference in downregulation activity among the H1, U6 Pol III promoters and the RSV Pol II promoter. However, a significant difference in GFP downregulation activity was observed between shRNA and miRshRNA configurations using the U6 promoter, suggesting that there is likely a difference in RNA processing efficiency between these two configurations to generate functional siRNA.

For RRVs expressing both yCD2 and miRshRNA, the decrease in yCD2 expression in RRV-RSV-yCD2 compared to RRV-yCD2 in which the yCD2 protein expression is mediated by IRES is probably due to promoter interference between the viral MLV and RSV LTR promoters as indicated by slightly slower replication kinetics of RRV-RSV-yCD2 compared to that of RRV-yCD2. It is unclear why the promoter interference has less effect on RRV-RSV-miRPDL1. On the other hand, the further decrease of yCD2 protein expression and the decrease in PDL1 downregulation activity seen in RRV-RSV-yCD2-miRPDL1 compared to RRV-RSV-yCD2 and RRV-miRPDL1 is probably attributable to additional factors other than promoter interference. One possible explanation is the mutually exclusive cellular compartmental allocation of the RSV-mediated transcript for miRNA processing and for yCD2 protein translation. A similar phenomenon was seen in RRV-RSV-yCD2-U6-miRPDL1 bearing two internal promoters (RSV and U6), except the PDL1 downregulation activity was significantly compromised. Another possible explanation could be limited accessibility of the RNA Pol II and the RNA Pol III polymerase complexes to their designated promoter. Although the two RNA polymerase complexes do not share the same transcriptional factors, steric hindrance issues may be responsible as the RSV-yCD2 and U6-miRPDL1 cassettes are located within 700 bp in proximity. Therefore, the drastic loss of PDL1 downregulation activity seen in RRV-RSV-yCD2-U6-miRPDL1 may be caused by a physical hindrance between the two RNA polymerase complexes in addition to vector instability as indicated by emergence of a deletion mutant.

One potential configuration we have not attempted to pursue is an RRV containing multiple miRshRNA cassettes. Although construction of multiple shRNA or miRshRNA cassettes have been reported in various expression vectors,[17,22,39,40] incorporating such a design in an RRV genome could be technically challenging due to the repeated sequence in miRNA30-derived backbone in the same vector that may lead to early emergence of deletion mutants.

In conclusion, we present here multiple configurations of RRVs that allow miRshRNA expression and potent downregulation activity of targeted genes involved in suppression of anti-tumor immunity. Delivery of checkpoint inhibitors, such as RRV-miRPDL1, may provide an opportunity to target immune checkpoints from within tumors that are less or unresponsive to conventional approaches such as monoclonal antibodies. In addition, this approach seems likely to lead to an improved toxicity profile, as the treatment is cancer-selective and localized within the tumor microenvironment. The RRV-miRPDL1 approach may be used alone or in combination with other therapeutic agents to help activate the immune system against tumors, especially given the local immune activity safety profile.

MATERIALS AND METHODS

Cell Culture

293T cells were obtained through a materials transfer agreement with the Indiana University Vector Production Facility and Stanford University deposited with ATCC (SD-3515; lot no. 2634366). Human glioblastoma cell lines U87-MG (ATCC, HTB-14), LN-18 (ATCC, CRL-2610), PC-3 (ATCC, CRL-1435), MDA-MB-231BR, a brain-seeking sub-line of human breast cancer cell line MDA-MB-231 (a kind gift from Dr. Noriyuki Kasahara at University of Miami), and 293T cells were cultured in complete DMEM medium containing 10% FBS (Hyclone), sodium pyruvate, GlutaMAX (Thermo Fisher Scientific), and antibiotics at penicillin 100 IU/mL and streptomycin 100 IU/mL (Corning). Human melanoma cell lines Mel103 (a kind gift from Dr. Bernard A. Fox at Oregon Health & Science University) were cultured in completed RPMI medium containing 10% FBS (Hyclone), GlutaMAX (Thermo Fisher Scientific), and antibiotics at penicillin 100 IU/mL and streptomycin 100 IU/mL (Corning).

Construction of RRV-shGFP, RRV-miRGFP, RRV-shGFP, RRV-miRPDL1, and RRV-yCD2-miRPDL1 Plasmid DNA

RRVs derived from pAC3-yCD2[41] containing human H1, U6 Pol III, or RSV Pol II promoter were generated to express conventional 21-nucleotide stem shRNA (shGFP and shPDL1) or microRNA30-derived shRNA with a 21-nucleotide stem as described[37] against GFP (miRGFP), human *PDL1* (miRPDL1), human *IDO-1* (miR-IDO1), or human *TGF-β2* (miRTGFb2) (Table S4 for sequences tested). RRVs expressing yCD2 and miR-PDL1 were designed to express both yCD2 and miRPDL1 from the RSV promoter (RRV-RSV-yCD2-miRPDL1) or from the RSV and U6 promoter separately (RRV-RSV-yCD2-U6-miR-PDL1). Each promoter-shRNA or promoter-miRshRNA cassette contains a restriction enzyme site Mlu I at the 5′ end and a Psi I at the 3′ end for direct replacement of the corresponding IRES-yCD2 cassette in the pAC3-yCD2 backbone (Figure 1). Sequences of all synthesized DNA fragments were confirmed by sequencing prior and post cloning into the RRV backbone.

Virus Production, Titer, and Infection

Virus stock was first produced by transient transfection of 293T cells using the calcium phosphate precipitation method.[23] Viral titer from transiently transfected 293T cells was performed by qPCR as described.[41] Viral infection was performed at a MOI of 0.1 in LN-18 cells and MOI of 1 in MDA-MB-231BR cells.

Viral Replication Kinetics by qRT-PCR

Viral replications were measured by qRT-PCR to quantify the amount of viral RNA present in supernatant over the course of infection. LN18 ells were seeded at 2×10^5 cells/well/2 mL of 6-well plate 1 day prior to infection. The next day, virus supernatants collected from transient transfection in 293T cells were used to perform infection at a MOI of 0.1. Supernatants from infected cells were collected every 3 days during the course of infection, filtered through a 0.45 μM syringed filter, and stored at −80°C. At each collection time point, the cells number were counted and passaged at 1:8 cells in a total volume of 2 mL to a new 6-well plate. Twenty microliters of all viral supernatants that were used to extract viral RNA using the Promega Maxwell 16 system (Promega, cat. no. AS1270). The eluted RNAs were centrifuged at 14,000 rpm for 1 min to remove magnetic beads carried over in the extraction process, supernatants were subsequently transferred to a 0.2 mL tube and stored at −80°C.

Viral RNA purified from Toca 511 to generate a standard curve was performed in parallel with experimental samples as described.[42] Purified RNAs were analyzed using one-step RT-qPCR with the Env2 primer set: Env2-F (5′-ACCCTCAACCTCCCC TACAAGT-3′); Env2-R (5′-GTTAAGCGCCTGATAGGCTC 3′); and probe (5′-TEX615/-AGCCACCCCCAGGAACTGGAGATAGA/3′IAbRQSp). RT-qPCR was performed using qScript XLT One-Step RT-qPCR ToughMix ROX (Quanta, cat. no. 95133-500) in a total reaction volume of 20 μL. Control reactions without RT were performed using iQ Supermix (Bio-Rad, cat. no. 170-8864). The RNA titer reported in copy/mL is determined from a six-log serial dilution RNA standard curve and without taking into the account of duplex viral RNA genomes per viral particle.

Selection of shGFP, miRGFP, shPDL1, and miRPDL1 with High Level of Downregulation Activity

U87-MG cells expressing GFP (U87/GFP) were generated by transduction with lentiviral vector (LV-CMV-GFP) at an MOI of 5 to achieve nearly 100% transduction and high GFP expressing level and U87/GFP cells were infected with RRV-shGFP or RRV-miR-PDL1 at a MOI of 0.1, 1, or 10. GFP expression level was monitored over a period of 30 days post infection and the GFP downregulation activity was quantified by flow cytometric analysis (see below) and compared to uninfected U87/GFP cells.

LN-18 cells, which have detectable basal PDL1 cell surface expression and can be further upregulated by IFNγ (Figure S3), were used for

selecting desirable shPDL1 and miR-PDL1 constructs. Cells were infected with RRV-shPDL1 or RRV-miRPDL1 at a MOI of 0.1. PDL1 cell surface expression was measured at day 14 post infection at which time maximal infectivity has been reached. PDL1 upregulation by IFNγ was performed in maximally RRV-shPDL1 and RRV-miR-PDL1 infected cells by adding 500 IU/mL recombinant human IFNγ (R&D System, cat. no. 285-IF-100/CF) for 36 hr before harvesting the cells for flow cytometric analysis.

Quantification of *PDL1* and *OAS1* RNA Expression by qRT-PCR

RNA was extracted from infected cells using the RNeasy Kit with DNase I treatment (Qiagen). Reverse transcription was carried out with 100 ng total RNA using a High Capacity cDNA Reverse Transcription Kit (ABI). qPCR was performed using TaqMan Universal PCR Master Mix, No AmpErase UNG (ABI, P/N no. 4324018) and TaqMan gene expression primers (ABI), (ID no. Hs01125301_m1 for *PDL1*, ID no. Hs00973637_m1 for *OAS1*, and Hs99999905_m1 for *GAPDH*) according to manufacturer's protocol. *PDL1* relative expression is calculated using the $2^{-\Delta\Delta\ (Ct)}$ method, with respect to expression in uninfected cells and converted to percentage. Experiment in which U87-MG cells were treated with exogenous recombinant human IFNα2a (PBL Interferon Source, product no. 11100-1, at 200 U/mL), the treatment was applied to the culture medium approximately 16 hr prior to cell harvest for RNA extraction.

Quantification of GFP and PDL1 Protein Expression Levels by Flow Cytometry

RRV-shGFP and RRV-miRGFP infected U87-MG/GFP cells were harvested at the time which maximal infectivity has been reached. GFP protein expression levels were quantified by measuring mean fluorescence intensity (MFI). The MFI of uninfected U87-MG/GFP cells was set to 100% and used to quantify the relative percentage of GFP expression in the RRV-shGFP and RRV-miRGFP infected U87/GFP cells.

RRV-shPDL1 or RRV-miRPDL1 infected cells were harvested using cell dissociation buffer (10 mM EDTA/PBS) and washed with autoMACS Rinsing Solution containing 10% BSA (Milteny Biotec, cat. no. 130-091-222 and 130-091-376). Cell pellets were resuspended in 100 μL autoMACS Rinsing Solution with isotype control (eBioSciences, cat. no. 12-4714) or anti-human PDL1-PE-conjugated antibody (eBioSciences, cat. no. 12-5983) according to the manufacturer's protocol. Stained cells were washed with autoMACS Rinsing Solution and resuspended in 600 μL autoMACS Rinsing Solution for flow cytometric analysis. PDL1 cell surface expression was quantified by subtracting the MFI value from isotype control, and the relative expression level was quantified by setting the MFI of IFNγ-treated, but uninfected cells to 100%.

Immunoblot

Maximally infected LN-18 cells were harvested and lysed for immunoblotting. Equal amounts of proteins from lysates were resolved on Criterion XT Precast Gel 4%–12% Bis-Tris Gels (Bio-Rad, cat. no. 345-0124). Mouse anti-human GAPDH (Millipore, cat. no. MAB374) antibody at 1:500 dilution was used to detect the expression of GAPDH, and mouse anti-yCD2 (Tocagen, clone 9A10F1) antibody at 1:1,000 dilution was used to detect the expression of yCD2 protein. Detection of protein expression was visualized using Clarity Western ECL Substrate (Bio-Rad, cat. no. 170-5060). Quantification of yCD2 protein expression level was obtained using the Quantity One Image Software version 4.6.7 (Bio-Rad). Ratios of measured GAPDH pixel density relative to U87-MG cell infected with IRES-yCD2 were used to normalize yCD2 expression levels.

Vector Stability Assay

Vector stability was measured from genomic DNA of maximally infected U87-MG cells after one round of infection at a MOI of 0.1 in U87-G or LN-18 cells. PCR was performed using the following primers: 5–127 (forward): 5′-CTGATCTTACTCTTTG-GACCTTG-3′ and 3–37 (reverse): 5′-CCCCTTTTT CTGGAGAC-TAAATAA-3′. The PCR cycling parameters were 94°C 2 min; followed by 30 cycles of 94°C 15 s; 62°C 30 s; and 72°C 1 min. Approximately 50 ng of genomic DNA and SuperTaq Polymerase (Thermo Fisher Scientific, cat. no. AM2052) were used for PCR. One-fifth of the PCR reaction was loaded on 1% agrose gel for electrophoresis analysis.

Trans-suppression of PHA-Stimulated Human PBMC by Co-culturing with PDL1 Expressing Tumor Cells

Cryopreserved PBMCs were collected anonymously from healthy volunteer donors who were Tocagen employees with oversight provided by the Tocagen Safety Committee. Dissociated cells from various non-donor-matched (allogeneic) tumor cell lines were treated with 50 μg/mL mitocycin C (Sigma-Aldrich, cat. no. M7949) at 37°C for 30 min followed by three washes with PBS to remove mitomycin C. Co-culture of tumor cells (2.5×10^5) and PBMC (5×10^5) treated with 1 or 10 μg/mL PHA (Sigma-Aldrich, cat. no. L1668) were seeded in 48-well plates in a final volume of 600 μL AIM V per well (Thermo Fisher Scientific, cat. no. 12055083) in the presence of 10% human serum AB (Sigma, cat. no. H4522-100mL) and 600 IU/mL recombinant human IL-2 (Proleukin from Prometheus Laboratories) for 72 hr. Approximately 8–10 hr prior to performing intracellular cytokine staining, 0.5 mL supernatant from stimulated co-cultured cells were removed for IFNγ ELISA and cells were replenished with equal volumes of fresh medium followed by treatment with 1 μL of Golgi Plug (BD Biosciences, cat. no. 555029). In some experiments, blocking antibody against PDL1 (clone 29E.2A3, BioLegend) and PD1 (clone EH12.1, eBioscience) were added to some wells at 10 μg/mL at the time of PHA stimulation.

ELISA and Intracellular Staining of IFNγ and TNF-α in PBMCs

IFNγ ELISA was performed using the human IFNγ ELISA set (BD Biosciences, cat. no. 555142) according to manufacturer's protocol. Co-staining of human IFNγ and TNF-α in PBMCs were performed using PE-conjugated anti-IFNγ (clone 4S.B3, eBiosciences, cat. no. 12-7319-82) and APC-conjugated anti-TNF-α (BD Biosciences, cat. no. 554514) antibodies according to manufacturer's recommendation.

SUPPLEMENTAL INFORMATION

Supplemental Information includes six figures and four tables and can be found with this article online at http://dx.doi.org/10.1016/j.omtn.2016.11.007.

AUTHOR CONTRIBUTIONS

A.H.L., D.J.J., C.G.T., and H.E.G. conceptualized the project; A.H.L. and D.J.J. wrote the manuscript; and A.H.L., C.G.T., R.B., A.H., L.A.M., and F.L.E. conducted the experiments.

CONFLICTS OF INTEREST

A.H.L., C.G.T., R.B., A.H., L.A.M., F.L.E., H.E.G., and D.J.J. are or were full time employees of Tocagen and hold stock or stock options in Tocagen.

ACKNOWLEDGMENTS

We thank Dr. Asha Das, John Wood, Dr. Nicholas A. Boyle, and Dr. Victoria Cohan for critical reading of the manuscript. The original RRV system was developed with support from NIH grants U01-NS59821 and R01-CA105171 to N. Kasahara. The work was also supported by Accelerate Brain Cancer Cure, American Brain Tumor Association (Washington, DC), Musella Foundation (Hewlett, NY), National Brain Tumor Society (Watertown, MA), Voices Against Brain Cancer (New York, NY), and the US Department of Health and Human Services (Washington, DC).

REFERENCES

1. Ostertag, D., Amundson, K.K., Lopez Espinoza, F., Martin, B., Buckley, T., Galvão da Silva, A.P., Lin, A.H., Valenta, D.T., Perez, O.D., Ibañez, C.E., et al. (2012). Brain tumor eradication and prolonged survival from intratumoral conversion of 5-fluorocytosine to 5-fluorouracil using a nonlytic retroviral replicating vector. Neuro-oncol. *14*, 145–159.
2. Cloughesy, T.F., Landolfi, J., Hogan, D.J., Bloomfield, S., Carter, B., Chen, C.C., Elder, J.B., Kalkanis, S.N., Kesari, S., Lai, A., et al. (2016). Phase 1 trial of vocimagene amiretrorepvec and 5-fluorocytosine for recurrent high-grade glioma. Sci. Transl. Med. *8*, 341ra75.
3. Bartlett, D.W., and Davis, M.E. (2006). Insights into the kinetics of siRNA-mediated gene silencing from live-cell and live-animal bioluminescent imaging. Nucleic Acids Res. *34*, 322–333.
4. Sioud, M. (2015). RNA interference: mechanisms, technical challenges, and therapeutic opportunities. Methods Mol. Biol. *1218*, 1–15.
5. Davidson, B.L., and McCray, P.B., Jr. (2011). Current prospects for RNA interference-based therapies. Nat. Rev. Genet. *12*, 329–340.
6. Deng, Y., Wang, C.C., Choy, K.W., Du, Q., Chen, J., Wang, Q., Li, L., Chung, T.K., and Tang, T. (2014). Therapeutic potentials of gene silencing by RNA interference: principles, challenges, and new strategies. Gene *538*, 217–227.
7. Sarisozen, C., Salzano, G., and Torchilin, V.P. (2016). Lipid-based siRNA delivery systems: challenges, promises and solutions along the long journey. Curr. Pharm. Biotechnol. *17*, 728–740.
8. Baker, M. (2010). RNA interference: Homing in on delivery. Nature *464*, 1225–1228.
9. Bauer, M., Kinkl, N., Meixner, A., Kremmer, E., Riemenschneider, M., Förstl, H., Gasser, T., and Ueffing, M. (2009). Prevention of interferon-stimulated gene expression using microRNA-designed hairpins. Gene Ther. *16*, 142–147.
10. Kenworthy, R., Lambert, D., Yang, F., Wang, N., Chen, Z., Zhu, H., Zhu, F., Liu, C., Li, K., and Tang, H. (2009). Short-hairpin RNAs delivered by lentiviral vector transduction trigger RIG-I-mediated IFN activation. Nucleic Acids Res. *37*, 6587–6599.
11. Zeng, Y., and Cullen, B.R. (2003). Sequence requirements for micro RNA processing and function in human cells. RNA *9*, 112–123.
12. Zeng, Y., Wagner, E.J., and Cullen, B.R. (2002). Both natural and designed micro RNAs can inhibit the expression of cognate mRNAs when expressed in human cells. Mol. Cell *9*, 1327–1333.
13. Boudreau, R.L., Martins, I., and Davidson, B.L. (2009). Artificial microRNAs as siRNA shuttles: improved safety as compared to shRNAs in vitro and in vivo. Mol. Ther. *17*, 169–175.
14. Grimm, D., Streetz, K.L., Jopling, C.L., Storm, T.A., Pandey, K., Davis, C.R., Marion, P., Salazar, F., and Kay, M.A. (2006). Fatality in mice due to oversaturation of cellular microRNA/short hairpin RNA pathways. Nature *441*, 537–541.
15. McBride, J.L., Boudreau, R.L., Harper, S.Q., Staber, P.D., Monteys, A.M., Martins, I., Gilmore, B.L., Burstein, H., Peluso, R.W., Polisky, B., et al. (2008). Artificial miRNAs mitigate shRNA-mediated toxicity in the brain: implications for the therapeutic development of RNAi. Proc. Natl. Acad. Sci. USA *105*, 5868–5873.
16. Boudreau, R.L., and Davidson, B.L. (2012). Generation of hairpin-based RNAi vectors for biological and therapeutic application. Methods Enzymol. *507*, 275–296.
17. Motegi, Y., Katayama, K., Sakurai, F., Kato, T., Yamaguchi, T., Matsui, H., Takahashi, M., Kawabata, K., and Mizuguchi, H. (2011). An effective gene-knockdown using multiple shRNA-expressing adenovirus vectors. J. Control Release *153*, 149–153.
18. Schaser, T., Wrede, C., Duerner, L., Sliva, K., Cichutek, K., Schnierle, B., and Buchholz, C.J. (2011). RNAi-mediated gene silencing in tumour tissue using replication-competent retroviral vectors. Gene Ther. *18*, 953–960.
19. Silva, J.M., Li, M.Z., Chang, K., Ge, W., Golding, M.C., Rickles, R.J., Siolas, D., Hu, G., Paddison, P.J., Schlabach, M.R., et al. (2005). Second-generation shRNA libraries covering the mouse and human genomes. Nat. Genet. *37*, 1281–1288.
20. Boudreau, R.L., Monteys, A.M., and Davidson, B.L. (2008). Minimizing variables among hairpin-based RNAi vectors reveals the potency of shRNAs. RNA *14*, 1834–1844.
21. Sliva, K., and Schnierle, B.S. (2006). Stable integration of a functional shRNA expression cassette into the murine leukemia virus genome. Virology *351*, 218–225.
22. Shan, Z.X., Lin, Q.X., Yang, M., Deng, C.Y., Kuang, S.J., Zhou, Z.L., Xiao, D.Z., Liu, X.Y., Lin, S.G., and Yu, X.Y. (2009). A quick and efficient approach for gene silencing by using triple putative microRNA-based short hairpin RNAs. Mol. Cell. Biochem. *323*, 81–89.
23. Lin, A.H., Liu, Y., Burrascano, C., Cunanan, K., Logg, C.R., Robbins, J.M., Kasahara, N., Gruber, H., Ibañez, C., and Jolly, D.J. (2016). Extensive replication of a retroviral replicating vector can expand the A bulge in the encephalomyocarditis virus internal ribosome entry site and change translation efficiency of the downstream transgene. Hum. Gene Ther. Methods *27*, 59–70.
24. Lin, A.H., Burrascano, C., Pettersson, P.L., Ibañez, C.E., Gruber, H.E., and Jolly, D.J. (2014). Blockade of type I interferon (IFN) production by retroviral replicating vectors and reduced tumor cell responses to IFN likely contribute to tumor selectivity. J. Virol. *88*, 10066–10077.
25. Bridge, A.J., Pebernard, S., Ducraux, A., Nicoulaz, A.L., and Iggo, R. (2003). Induction of an interferon response by RNAi vectors in mammalian cells. Nat. Genet. *34*, 263–264.
26. Logg, C.R., Logg, A., Tai, C.K., Cannon, P.M., and Kasahara, N. (2001). Genomic stability of murine leukemia viruses containing insertions at the Env-3′ untranslated region boundary. J. Virol. *75*, 6989–6998.
27. Curran, M.A., Montalvo, W., Yagita, H., and Allison, J.P. (2010). PD-1 and CTLA-4 combination blockade expands infiltrating T cells and reduces regulatory T and myeloid cells within B16 melanoma tumors. Proc. Natl. Acad. Sci. USA *107*, 4275–4280.
28. Hamid, O., Robert, C., Daud, A., Hodi, F.S., Hwu, W.J., Kefford, R., Wolchok, J.D., Hersey, P., Joseph, R.W., Weber, J.S., et al. (2013). Safety and tumor responses with lambrolizumab (anti-PD-1) in melanoma. N. Engl. J. Med. *369*, 134–144.
29. Topalian, S.L., Drake, C.G., and Pardoll, D.M. (2012). Targeting the PD-1/B7-H1(PD-L1) pathway to activate anti-tumor immunity. Curr. Opin. Immunol. *24*, 207–212.
30. Haile, S.T., Dalal, S.P., Clements, V., Tamada, K., and Ostrand-Rosenberg, S. (2013). Soluble CD80 restores T cell activation and overcomes tumor cell programmed death ligand 1-mediated immune suppression. J. Immunol. *191*, 2829–2836.

31. Hellmann, M.D., Friedman, C.F., and Wolchok, J.D. (2016). Combinatorial cancer immunotherapies. Adv. Immunol. *130*, 251–277.

32. Vilgelm, A.E., Johnson, D.B., and Richmond, A. (2016). Combinatorial approach to cancer immunotherapy: strength in numbers. J. Leukoc. Biol. *100*, 275–290.

33. Hickey, M.J., Malone, C.C., Erickson, K.E., Gomez, G.G., Young, E.L., Liau, L.M., Prins, R.M., and Kruse, C.A. (2012). Implementing preclinical study findings to protocol design: translational studies with alloreactive CTL for gliomas. Am. J. Transl. Res. *4*, 114–126.

34. Brown, B.D., Sitia, G., Annoni, A., Hauben, E., Sergi, L.S., Zingale, A., Roncarolo, M.G., Guidotti, L.G., and Naldini, L. (2007). In vivo administration of lentiviral vectors triggers a type I interferon response that restricts hepatocyte gene transfer and promotes vector clearance. Blood *109*, 2797–2805.

35. Naik, S., and Russell, S.J. (2009). Engineering oncolytic viruses to exploit tumor specific defects in innate immune signaling pathways. Expert Opin. Biol. Ther. *9*, 1163–1176.

36. Goldgraben, M.A., Russell, R., Rueda, O.M., Caldas, C., and Git, A. (2016). Double-stranded microRNA mimics can induce length- and passenger strand-dependent effects in a cell type-specific manner. RNA *22*, 193–203.

37. Zeng, Y., Cai, X., and Cullen, B.R. (2005). Use of RNA polymerase II to transcribe artificial microRNAs. Methods Enzymol. *392*, 371–380.

38. Kharytonchyk, S.A., Kireyeva, A.I., Osipovich, A.B., and Fomin, I.K. (2005). Evidence for preferential copackaging of Moloney murine leukemia virus genomic RNAs transcribed in the same chromosomal site. Retrovirology *2*, 3.

39. Liu, Y.P., Haasnoot, J., and Berkhout, B. (2007). Design of extended short hairpin RNAs for HIV-1 inhibition. Nucleic Acids Res. *35*, 5683–5693.

40. Spanevello, F., Calistri, A., Del Vecchio, C., Mantelli, B., Frasson, C., Basso, G., Palù, G., Cavazzana, M., and Parolin, C. (2016). Development of lentiviral vectors simultaneously expressing multiple siRNAs against CCR5, vif and tat/rev genes for an HIV-1 gene therapy approach. Mol. Ther. Nucleic Acids *5*, e312.

41. Perez, O.D., Logg, C.R., Hiraoka, K., Diago, O., Burnett, R., Inagaki, A., Jolson, D., Amundson, K., Buckley, T., Lohse, D., et al. (2012). Design and selection of Toca 511 for clinical use: modified retroviral replicating vector with improved stability and gene expression. Mol. Ther. *20*, 1689–1698.

42. Twitty, C.G., Diago, O.R., Hogan, D.J., Burrascano, C., Ibanez, C.E., Jolly, D.J., and Ostertag, D. (2016). Retroviral replicating vectors deliver cytosine deaminase leading to targeted 5-fluorouracil-mediated cytotoxicity in multiple human cancer types. Hum. Gene Ther. Methods *27*, 17–31.

OMTN, Volume *6*

Supplemental Information

Retroviral Replicating Vector Delivery of miR-PDL1 Inhibits Immune Checkpoint PDL1 and Enhances Immune Responses In Vitro

Amy H. Lin, Christopher G. Twitty, Ryan Burnett, Andrew Hofacre, Leah A. Mitchell, Fernando Lopez Espinoza, Harry E. Gruber, and Douglas J. Jolly

Supplemental Table 1: Viral titers of RRV of various shRNA configurations produced from transiently transfected 293T cells.

RRV	Titer (TU/mL)	Stdev
**IRES-yCD2 (Toca 511)**	5.48E+06	1.25E+05
**IRES-GFP**	3.30E+06	1.43E+05
**RSV-yCD2**	1.92E+06	1.85E+05
**U6-shGFP1***	2.95E+06	9.69E+04
**U6-shGFP2***	2.11E+06	1.43E+05
**U6-miRGFP1***	2.64E+06	1.08E+05
**U6-miRGFP2***	2.63E+06	8.59E+04
**U6-miRPDL1***	3.99E+06	2.33E+05
**H1-miRPDL1**	5.10E+06	5.48E+05
**RSV-miRPDL1**	1.34E+06	5.85E+04
**RSV-yCD2-miRPDL1**	2.00E+06	1.81E+05
**RSV-yCD2-U6-miRPDL1**	1.71E+06	3.18E+05

*Described as shGFP1, shRGFP2, miRGFP1, miRGFP2, miRPDL1, miRPDL1 in the manuscript.

Supplemental Table 2: RRVs of various shRNA configurations targeting *IDO-1*, *PDL1* and *TGF-β2*

shRNA configuration	Target with Down-regulation Activity > 75% [1]			
	GFP*	IDO-1*	PDL1*	TGF-β2*
H1-shRNA	0/2	NA	NA	NA
U6-shRNA	0/2	0/4	0/4	0/4
U6-miRshRNA	1/2	2/4	2/4	2/4
H1-miRshRNA	NA	1/1	1/1	NA
RSV-miR shRNA	NA	1/1	1/1	NA
RSV-yCD2-miR shRNA	NA	0/1	0/1	NA
RSV-yCD2-U6-miR shRNA	NA	0/1	0/1	NA

1. Ratios of the number of sequences that showed > 75% down-regulation activity to the number of sequences tested.

* Matching siRNA sequences tested in different configurations for each targets

Supplemental Table 3: RRVs of various shRNA configurations targeting *PDL1*.

Vector Configuration	PD-L1		
	N	Down-regulation activity >50% [1]	Down-regulation activity >75% [2]
U6-shRNA *	4	3/4	0/4
U6-miR30shRNA *	4	4/4	2/4
H1-miR30shRNA *	1	1/1	1/1
RSV-miR30shRNA *	1	1/1	1/1
RSV-yCD2-miR30shRNA *	1	1/1	0/1
RSV-yCD2-U6-miR30shRNA *	1	0/1	0/1

1. Ratios of the number of sequences that showed > 50% down-regulation activity to the number of sequences tested.
2. Ratios of the number of sequences that showed > 75% down-regulation activity to the number of sequences tested.

* Matching siRNA sequences tested in different configurations for each targets

N = number of sequence tested

Supplemental Table 4: Antisense sequences targeting human *IDO-1*, *PDL-1* and *TGF-β2*

Target	Accession #/Clone ID	Antisense seq (5- to 3’)
IDO-1	NM_002164	
Seq 1		TTCATTAAATCAGTGCCTCCA
Seq 2		TTTGGGTTCACATGATCGTGG
Seq 3		TGTTTAGCAATGAACATCCAG
Seq 4		TTACTTTGATTGCAGAAGCAG
PDL-1	NM_014143	
Seq 1		TTTCACATCCATCATTCTCCC
Seq 2		TTGATGGTCACTGCTTGTCCA
Seq 3		TTTCACAGTAATTCGCTTGTA
Seq 4		TCAATTGTCATATTGCTACCA
TGF-β2	NM_003238	
Seq 1		TTAGATGGTACAAAAGTGCAG
Seq 2		AACAGCATCAGTTACATCGAA
Seq 3		TTCACAACTTTGCTGTCGATG
Seq 4		TAGTTTTCTGATCACCACTGG

Supplemental Figure 1

A

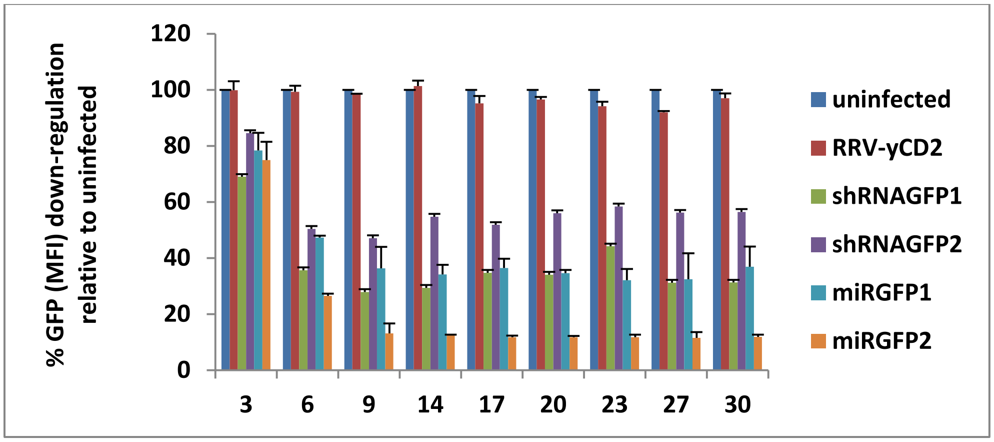

B

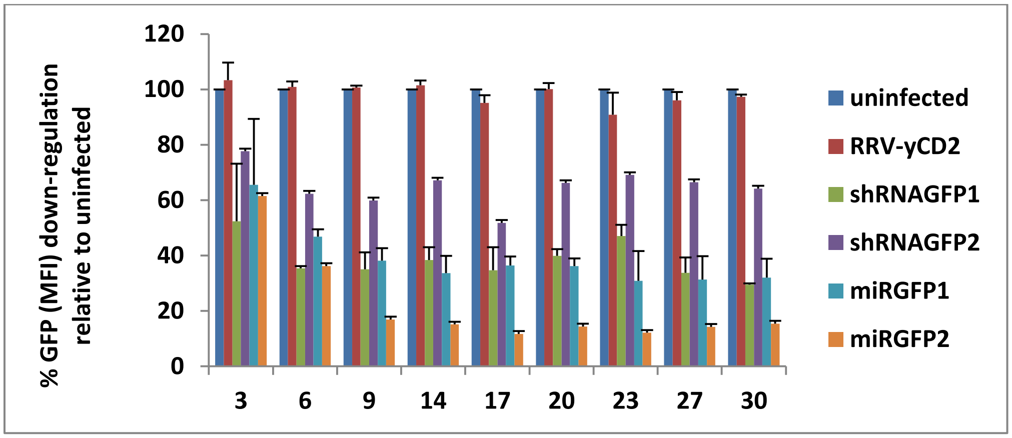

Figure S1: RRV-U6-miRGFP has higher GFP down-regulation activity than RRV-U6-shGFP. RRVs produced from transient transfection in 293T were used to infect U87-MG/GFP cells at MOI of 1 (A) or MOI of 10 (B). The GFP down-regulation activity was evaluated and quantified by flow cytometric analysis for mean fluorescent intensity (MFI). Both uninfected cells and cells infected with RRV-yCD2 were included as positive controls. The percentage of GFP down-regulation is relative to the uninfected cells. The data shown represent mean ± SD of triplicates performed from one of two independent experiments

Supplemental Figure 2

A

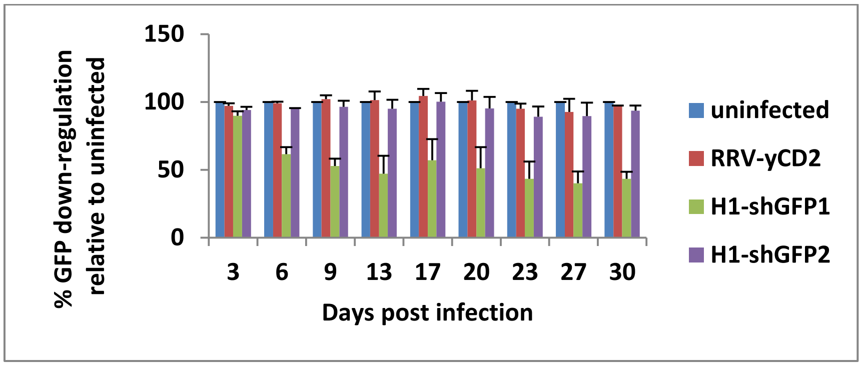

B

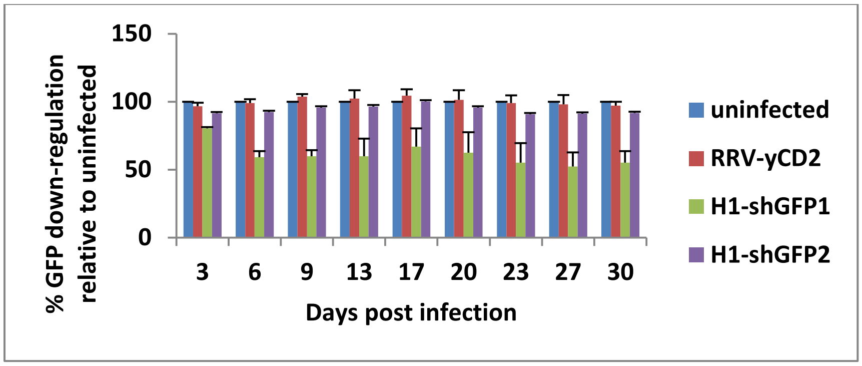

C

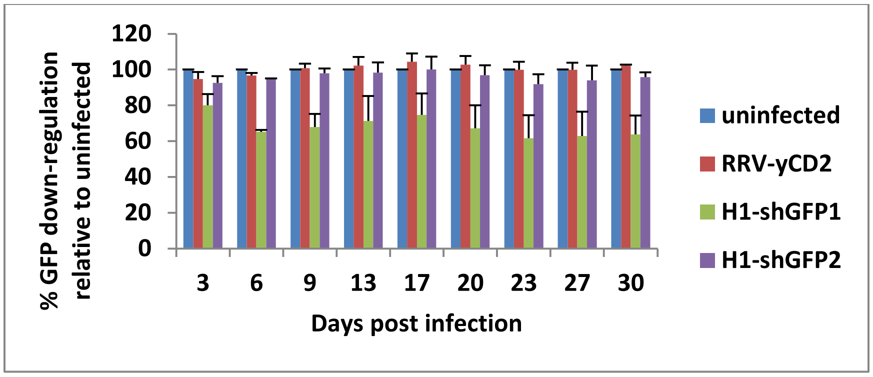

Figure S2: GFP down-regulation activity of RRV-H1-miRGFP RRVs produced from transient transfection in 293T were used to infect U87-MG/GFP cells at MOI of 0.1 (A), MOI of 1 (B) or MOI of 10 (C). The GFP down-regulation activity was evaluated and quantified by flow cytometric analysis for mean fluorescent intensity (MFI). Both uninfected cells and cells infected with RRV-yCD2 were included as positive controls. The percentage of GFP down-regulation is relative to the uninfected cells. The data shown represent mean ± SD of triplicates performed from one of two independent experiments.

Supplemental Figure 3

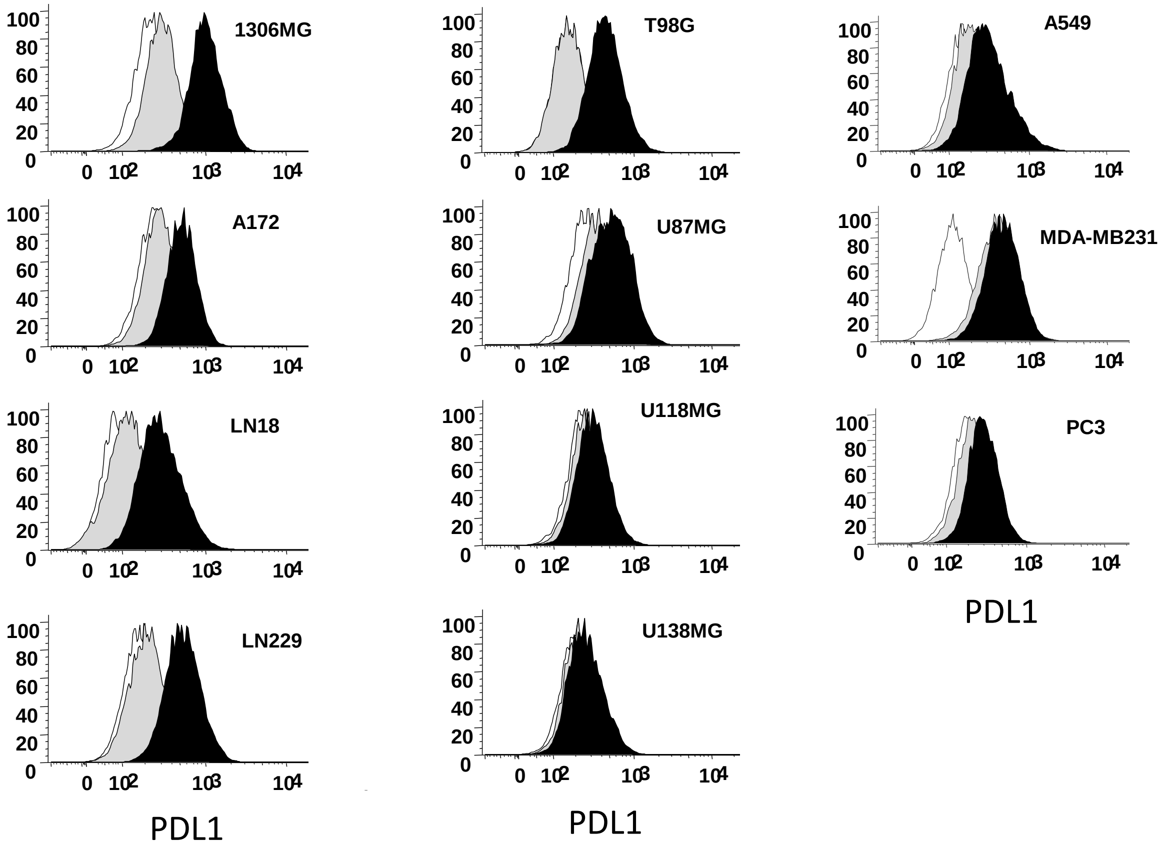

Figure S3: PDL1 cell surface expression in various tumor cell lines. Human glioblastoma cell lines 1306MG (a kind gift from Dr. Carol A Kruse), U87-MG, LN-18, LN-229, T98G, U118MG, U138MG, (ATCC), human lung carcinoma cells A549, human breast cancer cell line MDA-MB-231 and human prostate cancer cells PC-3 (ATCC) were cultured in complete culture medium containing 10% FBS (Hyclone), sodium pyruvate, GlutaMAX (Thermo Fisher Scientific), and antibiotics at penicillin 100 IU/mL, streptomycin 100 IU/mL (Corning). Cells treated with or without IFNγ at 500 IU/mL for 36 hours were harvested using cell dissociation buffer (10 mM EDTA/PBS) and washed with autoMACS rinsing solution containing 10% BS (Miltenybiotec, cat # 130-091-222 and cat # 130-091-376). Cell pellets were resuspended in 100 μL autoMACS rinsing solution with isotype control (eBioSciences, cat # 12-4714) or anti-human PDL1-PE-conjugated antibody (eBioSciences, cat # 12-5983) according to the manufacturer's protocol. Stained cells were washed with autoMACS rinsing solution and resuspended in 600 μL autoMACS rinsing solution for flow cytometric analysis. Isotype control (open histograms); basal PDL1 expression (gray filled histograms); and IFNγ-induced PDL1 expression (black filled histograms)

Supplemental Figure 4

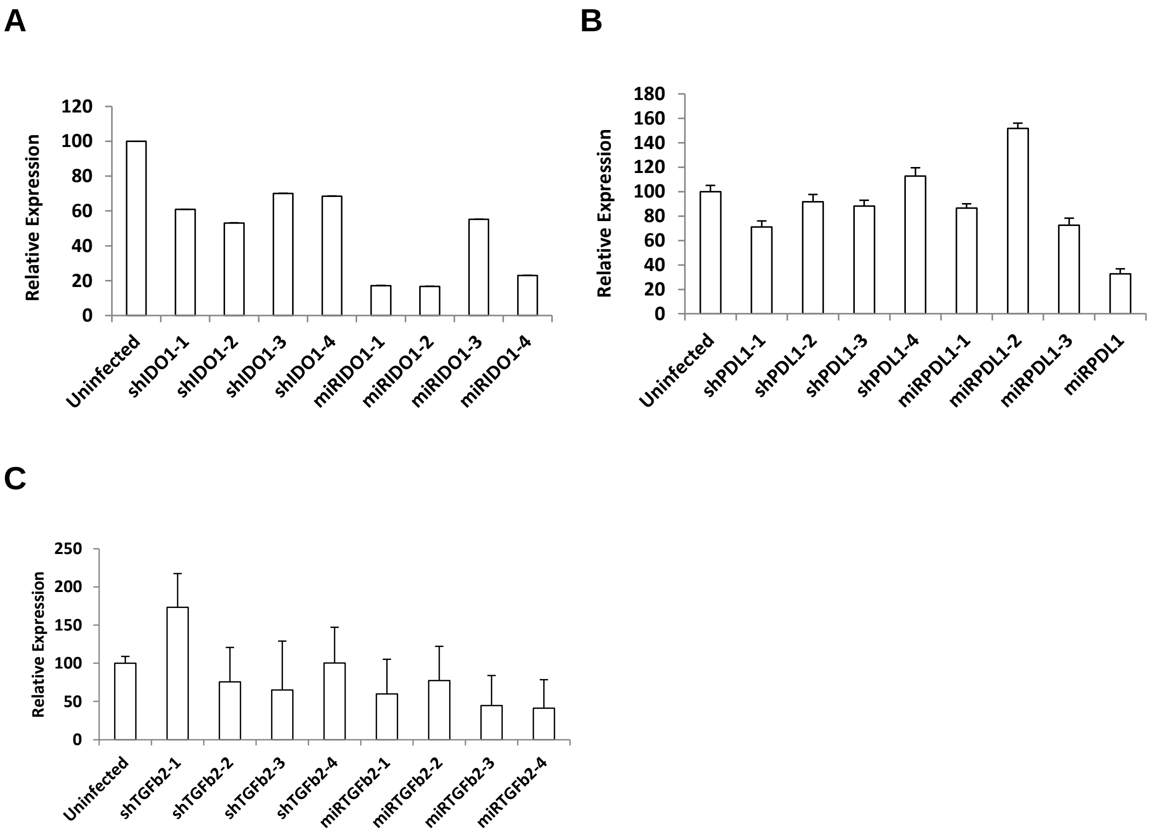

Figure S4: Quantification of *IDO-1*, *PDL1* and *TGF-β2* gene expression in LN-18 cells infected with RRVs expressing shRNA or miRshRNA. RNA was extracted from infected LN-18 cells using the RNeasy Kit with DNase I treatment (Qiagen). Reverse transcription was carried out with 100 ng total RNA using High Capacity cDNA Reverse Transcription Kit (ABI). Quantitative PCR (qPCR) was performed using TaqMan Universal PCR Master Mix, No AmpErase UNG (ABI, P/N # 4324018) and Taqman gene expression primers (ABI), (ID # Hs01125301_m1 for *PDL1*, ID# Hs 00984148_m1 for *IDO-1*, ID # Hs00234244_m1 for *TGF-β2*, and Hs99999905_m1 for *GAPDH*) according to manufacturer's protocol. Relative expression is calculated using the $2^{-\Delta\Delta(Ct)}$ method with respect to expression in uninfected cells and converted to percentage.

Supplemental Figure 5

A

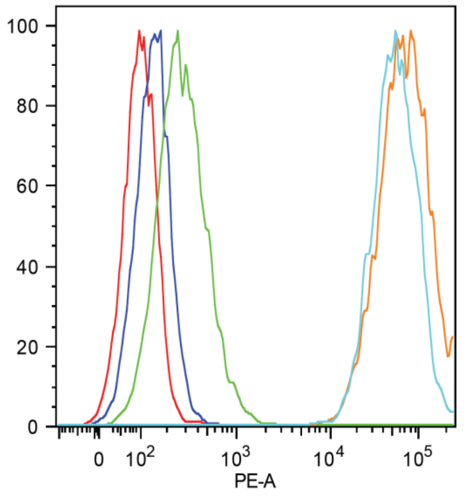

B

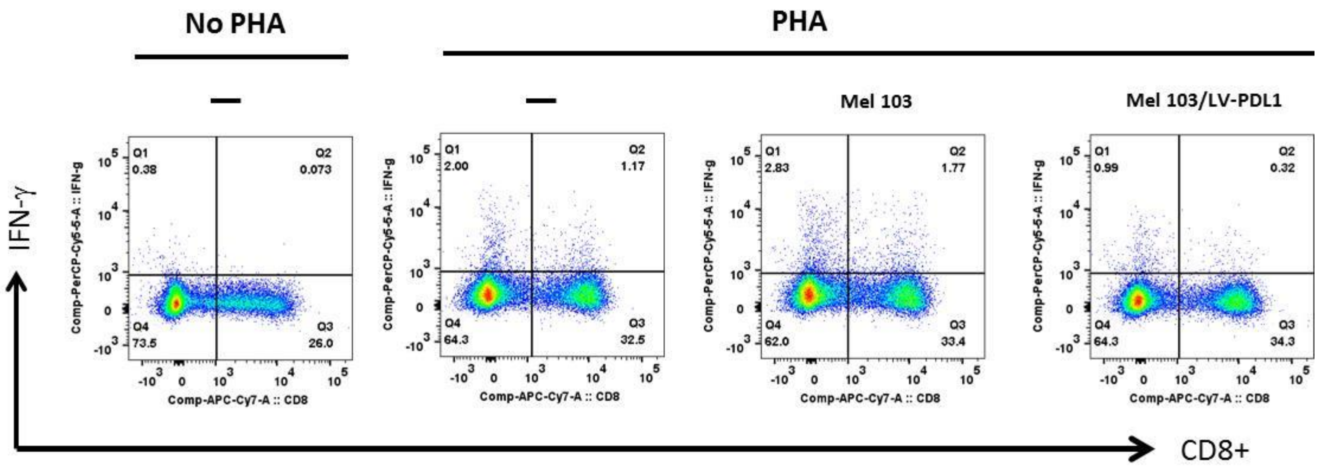

Figure S5: Cell surface expression of PDL1 in Mel 103 cells and Mel 103 cells overexpressing PDL1 (Mel103/LV-PDL1) in the presence or absence of IFNγ induction. Isotype control (red histogram); basal PDL1 expression (dark-blue histogram); IFNγ-induced PDL1 expression (green histogram) in Mel 103 cells. PDL1 overexpression (light-blue histogram) and IFNγ-induced (orange histogram) in Mel 103/LV-PDL1 cells. (B) PHA-induced PBMCs were cultured alone or cocultured with Mel 103 cells or Mel 103/LV-PDL1 cells. Intracellular IFNγ in CD8+ population with gating set based fluorescent minus one was analyzed by flow cytometry.

Supplemental Figure 6

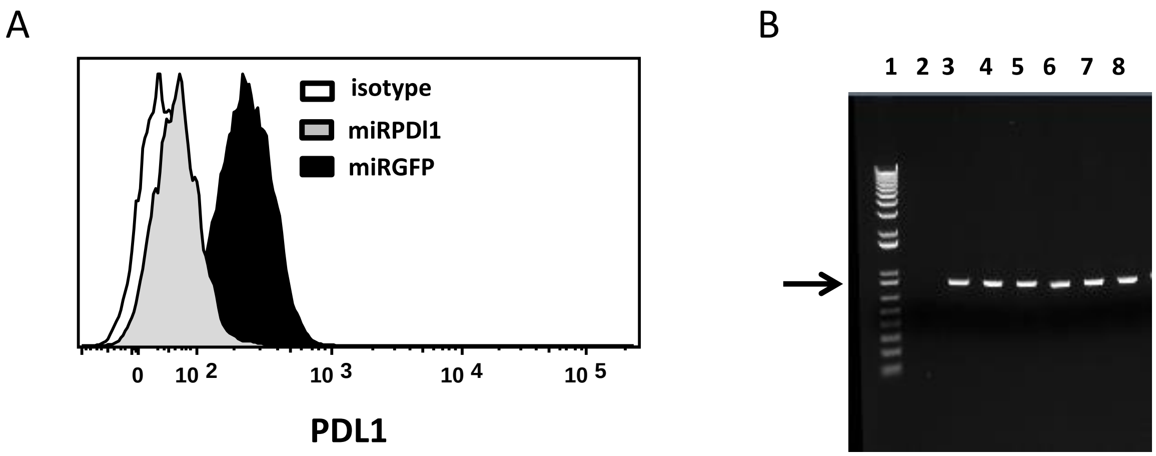

Figure S6: PDL1 down-regulation and vector stability of MDA-MB-231BR cells infected with RRV-miRPDL1. (A) MDA-MB-231BR cells infected with RRV-miRGFP and RRV-miRPDL1 were stained for PDL1 cell surface expression with PDL1 antibody and analyzed by flow cytometry. (B) Vector stability of RRV-miRGFP and RRV-U6-miRPDL1 infected MDA-MB-231BR cells was analyzed by end-point PCR 14 days post infection. Lane 1: DNA molecular marker (1kb plus marker, Invitrogen); lane 2, non-template control; lanes 3 and 6 are positive controls using the corresponding plasmid DNA as the templates; lane 4 (MOI of 0.1) and lane 5 (MOI of 1): RRV-miRGFP; lanes 7 (MOI of 0.1) and 8 (MOI of 1) : RRV-miRPDL1.
